# Affected Kindred Analysis of Human X Chromosome Exomes to Identify Novel X-Linked Intellectual Disability Genes

**DOI:** 10.1371/journal.pone.0116454

**Published:** 2015-02-13

**Authors:** Tejasvi S. Niranjan, Cindy Skinner, Melanie May, Tychele Turner, Rebecca Rose, Roger Stevenson, Charles E. Schwartz, Tao Wang

**Affiliations:** 1 McKusick-Nathans Institute of Genetic Medicine, Johns Hopkins University School of Medicine, Baltimore, Maryland 21205, United States of America; 2 Predoctoral Training Program in Human Genetics, Johns Hopkins University School of Medicine, Baltimore, Maryland 21205, United States of America; 3 Greenwood Genetic Center, Greenwood, South Carolina. 29646, United States of America; 4 Department of Genomic Sciences, University of Washington, Seattle, Washington 98195, United States of America; 5 Department of Pediatrics, Johns Hopkins University School of Medicine, Baltimore, Maryland 21205, United States of America; CNRS UMR7275, FRANCE

## Abstract

X-linked Intellectual Disability (XLID) is a group of genetically heterogeneous disorders caused by mutations in genes on the X chromosome. Deleterious mutations in ~10% of X chromosome genes are implicated in causing XLID disorders in ~50% of known and suspected XLID families. The remaining XLID genes are expected to be rare and even private to individual families. To systematically identify these XLID genes, we sequenced the X chromosome exome (X-exome) in 56 well-established XLID families (a single affected male from 30 families and two affected males from 26 families) using an Agilent SureSelect X-exome kit and the Illumina HiSeq 2000 platform. To enrich for disease-causing mutations, we first utilized variant filters based on dbSNP, the male-restricted portions of the 1000 Genomes Project, or the Exome Variant Server datasets. However, these databases present limitations as automatic filters for enrichment of XLID genes. We therefore developed and optimized a strategy that uses a cohort of affected male kindred pairs and an additional small cohort of affected unrelated males to enrich for potentially pathological variants and to remove neutral variants. This strategy, which we refer to as Affected Kindred/Cross-Cohort Analysis, achieves a substantial enrichment for potentially pathological variants in known XLID genes compared to variant filters from public reference databases, and it has identified novel XLID candidate genes. We conclude that Affected Kindred/Cross-Cohort Analysis can effectively enrich for disease-causing genes in rare, Mendelian disorders, and that public reference databases can be used effectively, but cautiously, as automatic filters for X-linked disorders.

## Introduction

X-linked Intellectual Disability (XLID) is a group of genetically highly heterogeneous disorders with mutations in genes on the X chromosome [1–3]. With the characterization of relatively common XLID genes, it is expected that the majority of the remaining mutations in unknown XLID genes are very rare and even private to individual patients and families [4]. Identification of these XLID genes is essential to provide accurate molecular diagnosis for individual XLID families and to better understand the molecular basis of intellectual function and disability in humans [2,3]. The extreme rarity and vast genetic heterogeneity of the individual XLID disorders pose a significant challenge, because ~10% of more than 1,000 annotated X-linked genes have already been implicated to cause half of all XLID disorders [1]. If these 10% of genes reflect the “low-hanging fruit”, then the remaining half of XLID cases without a known genetic cause will likely involve even more rare mutations affecting a broader range of genes, making them more difficult to isolate.

The rapid developments in high-throughput sequencing platforms that are coupled with effective targeted capture have made it possible to determine nearly all coding variants that present in an individual human genome [5–7]. Exome-based sequencing has become a powerful approach to elucidate the genetic basis of Mendelian disorders of unknown etiology and provide gene diagnoses of specific disorders with high genetic and phenotypic heterogeneity [8–11]. This strategy has had some success in identifying a handful of additional causes for autosomal intellectual disability [12,13]. The most significant challenge in using this strategy is in differentiating disease-causing (pathological) mutations from the large quantity of non-causal (neutral) variants. One may expect in any large scale exome sequencing study for approximately 20,000–24,000 variants to be found in an individual exome, with ~10%, coming from the X chromosome [6,8].

Common strategies for identifying causal mutations in rare Mendelian disorders include sequencing proband patients with same phenotype from multiple families [14,15], filtering out neutral variants using large databases such as dbSNP and the 1000 Genomes project [16–18], predicting functional relevance of variants using bioinformatics software such as SIFT [19] and PolyPhen-2 [20], conducting segregation analysis in proband families, and correlating known or predicted function of the candidate genes with the disease phenotype.

The success of these approaches relies on a number of factors: (1) the recruitment of multiple families with the same phenotype of interest, which can prove challenging for very rare Mendelian disorders with high locus heterogeneity and a wide spectrum of phenotypic expressivity; (2) the reliability of public variant databases, which are presumably generated from individuals lacking the disease under study, and would therefore only contain a pool of neutral variants for a given phenotype; (3) the reliability of bioinformatics tools in predicting variant significance; (4) a burden of pathological mutations in the functional portions of genes that are targeted for sequencing, like coding exons and splice sites, with less dependence on mutations occurring in poorly covered regions like regulatory elements; and (5) the extent of knowledge available on a candidate gene in mechanistically linking its biological functions to the phenotype.

To identify rare and causal mutations in heterogeneous X-linked Mendelian disorders, it is essential to utilize filters to remove the majority of neutral variants and sequencing errors in order to focus on potential pathological variants. Public databases such as dbSNP (http://www.ncbi.nlm.nih.gov/projects/SNP), the 1000 Genomes dataset (http://www.1000genomes.org), and the Exome Variant Server (EVS, http://evs.gs.washington.edu/EVS) have been used extensively as discrete variant filters for many studies [16–18,21]. The expected rarity of individual causal mutations in novel XLID makes it reasonable to eliminate common polymorphisms above a given frequency using data from these databases. Furthermore, it is generally assumed that public databases such as 1000 Genomes consist of individuals devoid of the phenotype of interest, and thereby serve as a public “normal control” set. The free and easy assess to these large “control” datasets makes them the top choices for many small scale genetic studies [16–18,21].

Our study aims to identify causal mutations in novel XLID genes using X chromosome exome sequencing. We systematically evaluated variant data from dbSNP, the 1000 Genomes, and EVS as discrete filters to determine how effectively each could reduce the number of neutral variants from our sequenced cohort. In doing so, we recognized that these databases present with limitations in their current forms as automatic filters to enrich for causal XLID genes. We therefore developed and optimized a strategy using affected male kindred pairs and affected unrelated males to enrich for potentially pathological variants and to remove neutral variants. Our study shows that this Affected Kindred/Cross-Cohort strategy achieves a substantial reduction in variants compared to the public database-dependent discrete filters alone. Importantly, our study shows that this strategy could achieve a significant enrichment for known and candidate XLID genes.

## Results

### Study Sample from X-Chromosome Exome Sequencing

**Fig 1:**
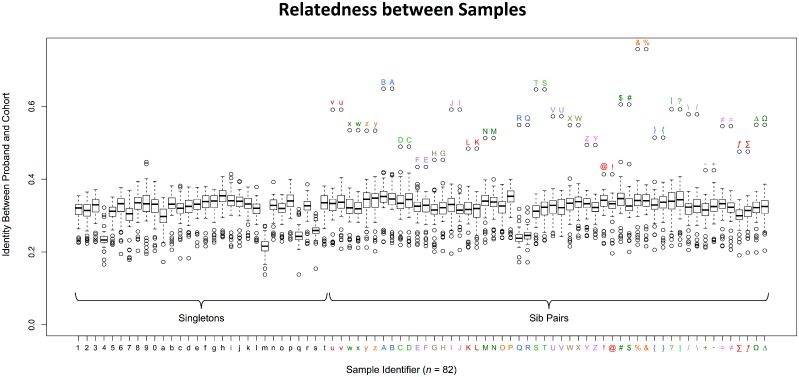
Relatedness between Study Samples. The estimated relatedness of samples is calculated by comparing the percentage of shared variants between two samples. The vertical axis shows the percentage of shared variants between two samples. The horizontal axis shows individual samples (n = 82) in this study cohort. Sporadic cases are samples that are not related to any other sample (left) while kindred pairs are two related affected males (right) (Table 1). The color-coded alphanumeric labels designate individual samples along the X-axis. There are 30 sporadic cases [1–9, 0, a-t; black labels] and 52 kinships (26 pairs) [u-z, A-Z, symbols; colored labels]. Box and whisker plots indicate overall identity between a proband and all other samples in the cohort. Identity ≈ (2 x # of variants identical between both samples) / (sum of variants of both samples). Sporadic cases generally share low identity with other samples. Paired kindred generally show the highest identity with each other. Paired kindred are juxtaposed with each other with the same color on the X-axis to simplify visualization of relationships. Outlier labels located above the hollow plots indicate the identifier for the sample that shares the highest identity, which is consistent with the known family relationship.

Genomic DNA samples from males with XLID (*n* = 82) were sequenced (**See Materials and Methods**). Of the 82 samples, 30 are single male probands from unrelated XLID families; the remaining 52 samples constitute 26 affected pairs including full brothers, maternal male cousins, and maternal uncle and nephew pairs (Table 1). The relationships of the affected pairs were validated by a calculation of the relatedness between samples in the entire study cohort (Fig. 1). Approximately 79.9% of the target regions are covered at ≥4x read depth across all samples with an average read depth of 49x. Variant calling on whole-genome aligned reads generated an average of 1,774 ± 239 variants per sample on or near target regions, and 14.7% of these variants are non-synonymous or are present at conserved splice junctions. Several discrete filters were then applied individually or cumulatively to enrich for potential causal variants. The total variant compositions prior to and after each filtering step are shown in Table 2 (Table 2).

**Table 1:** XLID Cohort for X Chromosome Exome Sequencing.

Relationship of Samples	Number (Pairs)
Affected Sporadic Cases	30
Affected Pairs	52 (26)
Brothers	44 (22)
Maternal Half-Brothers	4 (2)
Maternal Male First Cousins	2 (1)
Uncle-Nephew	2 (1)

All samples are diagnosed with an X-linked Intellectual Disorder. Criteria for X-linkage are described in **Materials and Methods**.

**Table 2:** Enrichment of Potential Pathological Variants in X-Exome of XLID Cohort with Different Variant Filters.

Application of Variant Filters	Non-Synonymous or Splicing Variants	Other Variants	Total Variants	% Original
Strand and Proximity Pre-filters Only ^1^	221.8 ± 30.8	724.5 ± 137.0	946.3 ± 167.8	100.0%
+ Shared Segment Filter ^2^	160.1 ± 65.0	511.3 ± 222.1	671.4 ± 287.1	71.0%
+ [1000G] Male-162 Internal Exome Filter ^3^	62.5 ± 19.9	645.6 ± 129.2	708.1 ± 149.1	74.8%
+ Exome Variant Server (Male Only) Filter ^4^	18.8 ± 4.6	545.9 ± 108.8	564.7 ± 113.4	59.7%
+ “Non-clinical” dbSNP Filter ^5^	11.9 ± 5.4	48.8 ± 18.5	60.7 ± 23.9	6.4%
+ Affected Kindred/Cross-Cohort Filter ^6^	7.5 ± 2.4	25.4 ± 2.2	32.9 ± 4.6	3.5%
All Filters ^7^	2.1 ± 1.7	12.1 ± 10.0	14.2 ± 11.7	1.5%

Average number of variants remaining per sample after sequential or aggregate filtering steps.

^1^ Strand and Proximity Pre-Filters are applied universally on top of all other filters. The percent of variants remaining after a particular filter is relative to the variant output after application of the Strand and Proximity Pre-Filters and is provided in column 5.

^2^ Shared Segment Filter: for demonstration purposes, results of this filter are provided separately from the rest of the Affected Kindred/Cross-Cohort Filter.

^3^ [1000G] Male-162 Internal Exome Filter: removes variants from the XLID cohort shared in common with 162 males from the 1000 Genomes.

^4^ Exome Variant Server (Male Only) Filter: removes variants from the XLID cohort shared in common with variants of the male fraction of EVS.

^5^ “Non-Clinical” dbSNP is redacted of known, probable, or potentially pathological variants in dbSNP Build 137.

^6^ Affected Kindred/Cross-Cohort Filter: results exclude the Shared Segment Filter component (see Row 2).

^7^ All filters, including re-introduction of known rare pathological variants (from dbSNP) that are inappropriately eliminated by the Affected Kindred/Cross-Cohort Filter.

### Assessment of Relatedness between Samples and Population Stratification

The relationships between affected pairs were validated by estimating relatedness (see **Materials and Methods**) across samples in the entire study cohort (Fig. 1). Additionally, relatedness of samples was assessed by ascertainment of regions of IBD (shared segments of genotypes inherited Identical By Descent) between all samples, using a combination of an automated 5 MB sliding window detector across IBD genotypes and by manual curation (S1 Fig.). By this process, no IBD sharing was detectable between samples known to be unrelated. For a more refined analysis of genetic sharing, we plotted the correlation of known linked SNPs to genotype sharing (S2 Fig.). SNPs that are known to be in linkage were found to never cross into boundaries defined as regions of IBD from S1 Fig. This suggests that recombination sites consistent with known linkage intervals define the boundaries for shared segments between related samples in our cohort. However, only higher resolution, full chromosome genotyping (not exome sequencing) can prove this conclusively. Based on these analyses, an estimation of relatedness across all sample genotypes, detection of regions of IBD across all samples, and definition of these IBD regions around known linked SNPs, the expected relatedness between samples is likely accurate.

Additionally, we looked for population stratification, in the event that large background population deviation may influence downstream analysis, particularly when using some public database-dependent filters. All samples in our cohort are expected to be of European descent. We compared the degree of deviation of individual samples from the cohort genotype mean, as well as the load of SNPs at high frequency in the European American population, as obtained from EVS (S3 Fig.). As expected, the majority of samples clustered together with genotypes of primarily European ancestry. However, five samples showed slight deviations from the main cluster. When SNP loads were compared to EVS data from the African American population, the sample cluster deviation was reproduced, indicating that these five samples have a small, but detectable contribution of African ancestry. We do not believe this marginal stratification will affect downstream analysis, due in part to the limited degree of deviation, the large presence of both African and European ancestry populations in public databases, and our desire to find rare mutations, which are less likely to be affected by genetic background.

### Variant Filtering Using Strand and Proximity Metrics

**Fig 2:**
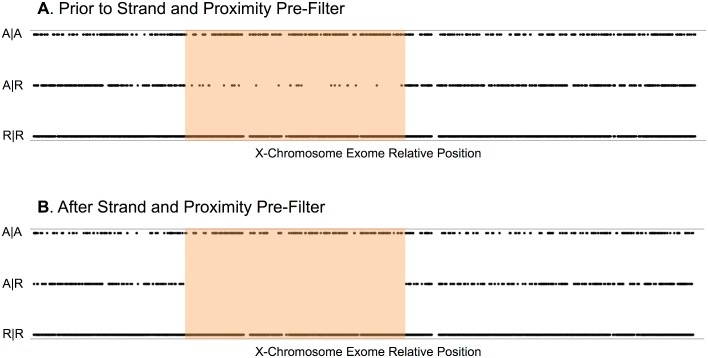
Shared Segment Filter and Error Reduction by Strand/Proximity Pre-Filter. The Shared Segment Filter (component of the Affected Kindred/Cross-Cohort Filter) retains chromosomal segments shared as Identical by Descent between two related samples in the XLID cohort. In this example, **Panels A** and **B** each reflect the same kindred pair, two brothers. The X-axis is position along the X chromosome exome. The Y-axis indicates the allelic status of a given variant for both siblings. Each point in the graph is a variant site for at least one sample. R|R allelic status indicates that the given point (genomic site) matches the reference sequence (hg19) in both samples (both samples are wildtype). A|A allelic status indicates the given point (variant site) is alternate to hg19 in both samples (both samples are hemizygous mutant). A|R allelic status indicates the given point matches reference in one sample and is alternate in the kindred sample (the samples are genotypically discordant). The orange blocks delineate chromosomal segments devoid of A|R points. All sequence in that segment is Identical by Descent between the two samples. The Shared Segment Filter retains variants (A|A) within the orange block. **Panel A** shows variant allele status in the Shared Segment Filter prior to the application of the strand- and proximity-based pre-filters. With the exception of the rare *de novo* mutation, there should be no discordant (A|R) variants within the orange block. Such variants are likely erroneous. **Panel B** shows the Shared Segment Filter after application of the strand- and proximity-based pre-filters. The A|R variants previously present in the orange block are eliminated, reflecting a reduction in erroneous variant calls as a result of these pre-filters.

We have previously observed that many false positive variant calls can be efficiently and specifically removed by applying two pre-filters determined by the proportion of base call from opposing strands and by inter-variant proximity [22]. Firstly, during read alignment, sequenced reads may be aligned to either the positive (Crick) or negative (Watson) strand. Variant base calls are therefore made in relation to either the positive or negative strand. An excess of variant base calls from one strand over another often characterizes false positive variants. The strand-based pre-filter eliminates variant calls that are not represented by at least one variant base call on each strand. Secondly, false positive variant calls often aggregate in close proximity. Generally, we should not observe more than two variants in a 1,000 bp stretch of DNA in a single individual [23]; even less should be observed for more evolutionarily conserved regions, like exons [24]. The proximity-based pre-filter very conservatively removes variant calls if they are present within 10 bp of each other. The consequence of these pre-filters is a substantial reduction in the frequency of erroneous variants that would otherwise confound downstream analysis (Fig. 2). Both of these pre-filters are applied universally, prior to any other filter method, described below (**Table 2, Row 1**).

### Variant Filtering Using dbSNP (Build 137)

To enrich potential disease-causing variants for further genetic and functional studies, we tested multiple filtering methods. Numerous published studies have made use of variants in dbSNP to filter out variants of non-clinical significance [14,15,25]. Build 137 of dbSNP includes variants of known pathological significance; we therefore selectively removed dbSNP variants that are present in the CLINVAR database (http://www.ncbi.nlm.nih.gov/clinvar; CLINSIG = 4 [probable-pathogenic] or 5 [pathogenic]). Prior removal of these variants of known pathological significance from dbSNP is important, as there may be overlap between these pathological variants and disease-causing variants in our study cohort. Retention of these pathological variants in dbSNP would result in their removal from our cohort, creating a false negative.

This abridged dbSNP dataset is still large and will likely successfully filter out many non-pathological variants. However, we expect using this method will be flawed to the extent that most pathological variants in dbSNP are not known nor annotated, and are therefore retained in this abridged dbSNP. Such unannotated pathological variants may overlap with important variants of interest in our study; such variants would be unintentionally filtered out, resulting in a higher false negative rate. To assess the fraction of remaining unannotated variants that may be potentially pathological, we identified the predicted truncating mutations (nonsense and frame-shift) that occur transcriptionally upstream of known deleterious mutations. Of the 27,242 coding variants present in our abridged dbSNP, 100 (or 0.37%) fit the above description. This is a highly conservative estimation, as it does not account for additional downstream nonsense and frame-shift mutations. Importantly, it does not account for missense and splicing changes that likely constitute a much larger fraction of remaining deleterious variants in known disease genes.

Given the rarity of XLID, potential XLID variants that are present but not annotated as pathological in dbSNP should have a very low minor allele frequency (MAF). Variants below a specified MAF could be removed from our abridged dbSNP filter, thereby ensuring we are not filtering out potentially pathological variants in our cohort. A frequent cutoff for rare variants is an MAF of 1%. However, we determined a more accurate MAF cutoff could be derived for male-restricted X chromosome variants of interest, because the majority of MAFs for dbSNP are derived from the 1000 Genomes Project. There are 525 male X chromosomes and 1,134 (2 x 567) female X chromosomes in 1000 Genomes (1,659 X chromosomes total). An appropriate MAF cutoff would minimize the probability that an unannotated pathological variant exists only in unaffected female carriers and never exists in unaffected males in 1000 Genomes. We calculated that if a variant is present in ≥12 of the 1,659 copies of the X chromosome (MAF ≤ 0.73%) in 1000 Genomes, the probability that all 12+ variant copies are present in only female carriers is ≤1% (S1 Table). We therefore chose to remove from our abridged dbSNP dataset all variants with a 1000 Genomes MAF ≤ 0.73%, to mitigate the unintended loss of unannotated pathological variants from our study cohort during filtration.

This new, “Non-Clinical” dbSNP Filter, redacted of known pathological variants and variants of low MAF, when used on our XLID cohort, results in a substantial 93.6% reduction in the number of variants for further analysis (**Table 2, Row 5**).

### Variant Filtering Using 1000 Genomes

Individuals in the 1000 Genomes came from ethnically diverse populations and are not expected to have severe intellectual disability. The master variant output for the 1000 Genomes project (Integrated Phase 1, version 3: 20101123) includes calls made from both males and females. Females could potentially possess an XLID mutation in the heterozygous state (carrier status) without clinical phenotype, while males are not expected to carry disease-causing mutations for XLID in the hemizygous state. We reason that variant data from males in the 1000 Genomes can be used as a filter to reduce the number of neutral variants in the X-exome sequenced from males in our XLID study. We thus generated a male-only variant dataset (*n* = 525) by removing all X chromosome variant calls made only in the female portion of the 1000 Genomes.

Publicly accessible variant data output from the 1000 Genomes consist of low coverage and exome variant calling including SNPs and INDELs at the current stage. This data was generated from multiple parallel pipelines at different sequencing and data analysis centers, and then merged into one VCF file [26]. For initial assessment of this dataset as a variant filter in our project, we analyzed the composition of variant genotypes for all the chromosomes from the 1000 Genomes project. Two unexpected discrepancies were noted in our analysis, which preclude the immediate use of the 1000 Genomes dataset as a filter.

Firstly, we observed the presence of ambiguous genotypes among male variant calls. An individual can have a genotype (sum of variant alleles at variant position) of 0 (no variant), 1 (heterozygous), or 2 (homozygous) as compared to the established reference. Because the X chromosome is in hemizygous state in males, we anticipate that genotypes for the male X chromosome should only be assigned as 0 or 1. However, we observed many ambiguous genotypes, including 2, 3, and 4, as well as non-integer genotypes ranging from 0.05 to 3.95 in a substantial fraction of variant calls (Table 3). Ambiguous genotypes account for ~9% of non-zero genotypes and have integer and non-integer values ranging from 0.05 to 4. A non-integer value for the number of alleles of a variant should not occur, with the exception of somatic mutations. The restricted presence of these ambiguous non-integer values to only male X chromosome variants, and the complete lack of such values among autosomal or female X chromosome variants, suggests that they are erroneous.

**Table 3:** Ambiguous Variant Calls in the Public 1000 Genomes Variant Dataset.

Sex	Chromosome	Genotype	Heterozygous	Homozygous	Ambiguous
		Variant Call	1	2	Others
Males	X Chromosome		39.49%	51.55%	8.96%
Females	X Chromosome		90.54%	9.46%	0%
Males	Autosomes		92.34%	7.66%	0%
Females	Autosomes		92.34%	7.66%	0%

Variant call = 1: Percent of variant alleles present as one copy in a sample (heterozygous state). Variant call = 2: Percent of variant alleles present as two copies in a sample (homozygous state). Variant call = Others: Percent of variant alleles present in copies other than 1 or 2, including non-integer counts. All values are evaluated exclusively from coding sequence variants for the respective chromosomes and sexes. Only the male X chromosome dataset possesses ambiguous genotypes. All variants were obtained from the 1000 Genomes variant dataset, pre-separated by chromosome [Integrated Phase 1, version 3: 20101123].

Secondly, we assessed the concordance between 1000 Genomes variant calls and SNP genotyping (Omni Platform), to approximate the accuracy of the variant output. Concordance for the X chromosome was calculated to be 68.6 ± 4.7%, which is much lower than expected [26]. To clarify this discrepancy, we generated variant calls directly from aligned sequence data of 162 random males in the 1000 Genomes using the Unified Genotyper and compared it to the Omni genotype data. Correlation between these 162 samples and their respective Omni genotypes was calculated at 98.8 ± 0.4%, which is comparable to what has been previously reported [26]. Given these apparent discrepancies, we have chosen not to use the current publicly accessible build of the 1000 Genomes as an automatic variant filter in our XLID project. Instead, we use the internally generated genotypes from the 162 male samples. This [1000G] Male-162 Internal Exome Filter is the least efficient discrete filter in our analysis, reducing the average variant count per sample by only 25.2% (**Table 2, Row 3**).

### Variant Filtering using the Exome Variant Server Dataset

The Exome Variant Server (EVS) dataset was generated through the NHLBI Exome Sequencing Project, by sequencing more than 5,000 exomes for samples collected as “healthy” controls or samples diagnosed with a heart, lung, or blood disorder. Samples with an XLID should be rare, if present at all. Approximately half of EVS samples are male. For the X chromosome, male genotypes for the non-pseudoautosomal regions can be distinguished from female genotypes, because male genotypes are explicitly annotated as hemizygous or wildtype. This EVS (Male Only) Filter, much like the [1000G] Male-162 Internal Exome Filter, is based on the assumption that none of the variants in these “control” populations will contribute to XLID, and can therefore be subtracted from the variant list in our XLID cohort.

This filter performs much better than using the [1000G] Male-162 Internal Exome Filter (40.3% reduction in variants), but does not outperform the dbSNP filter (**Table 2, Row 4**). For our analysis, this database is presumably the most reliable of the three tested, as it is consistent in its genotyping and the population in question should not have any overlapping disease traits with our analysis. However, we anticipate that the use of the EVS cohort, which is still a disease population (heart, lung, and blood disorders), will complicate other X-linked studies examining similar disease traits.

### Variant Filtering by Relatedness

The rarity of XLID and the distinct inheritance (no male-to-male transmission) predict that that the remaining XLID mutations are likely very rare and even private to individual families and are therefore unlikely to be shared with unrelated families (with rare exceptions) [4]. Based on this prediction, we have developed and implemented a filter based on relatedness, which we call the Affected Kindred/Cross-Cohort Filter.

One component of this filter, referred to as the Shared Segment Filter, retains all variants present within regions observed as shared (Identical by Descent) between affected related samples in the cohort (Fig. 2). This is a relatively simple step that results in a quick reduction in variants, while simultaneously providing confirmation of sample relatedness. When this step is applied alone, it results in a 29% reduction in potentially neutral variants (**Table 2, Row 2**).

**Fig 3:**
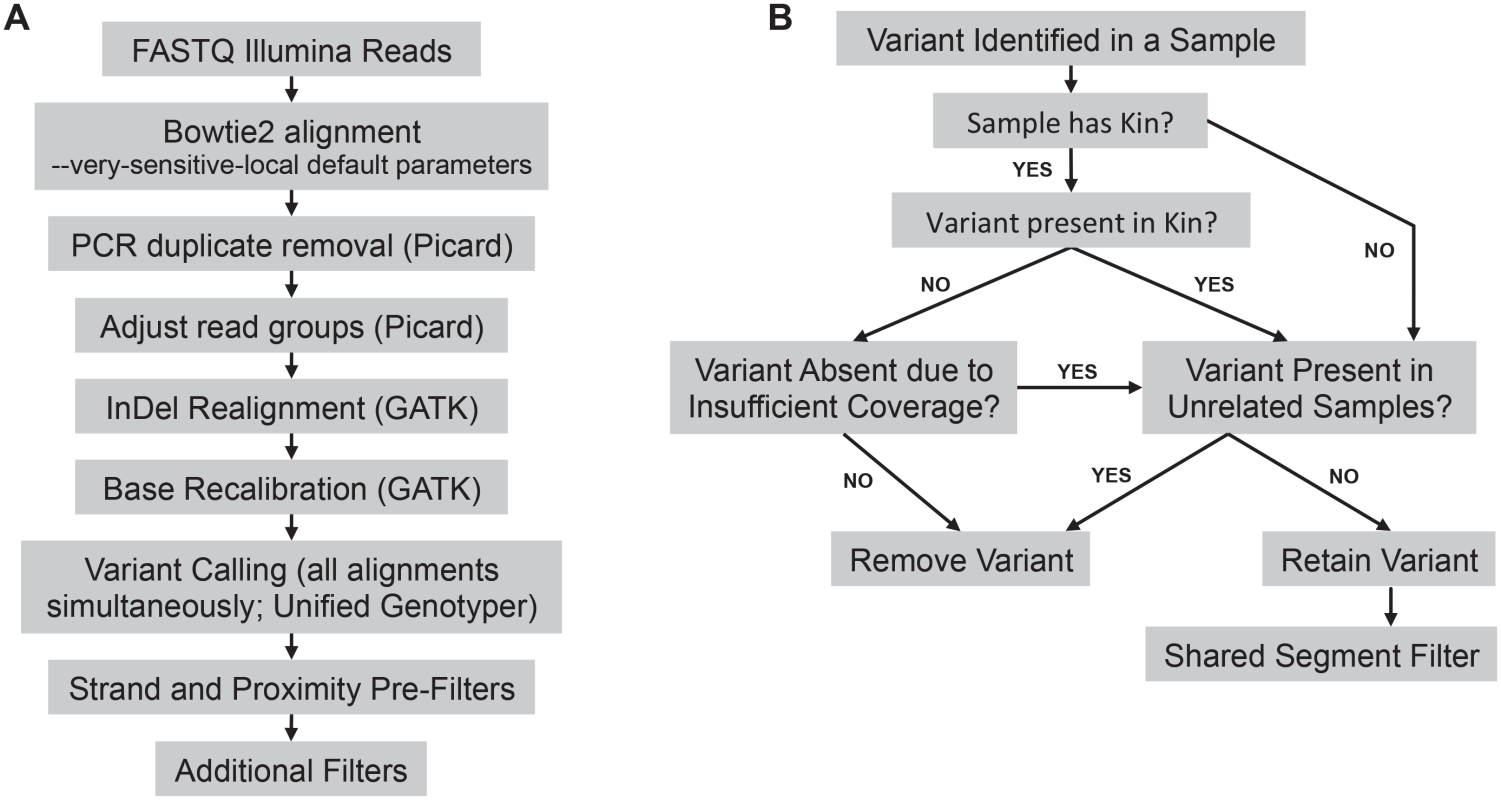
Schematics of Variant Calling and Affect Kindred/Cross-Cohort Analysis. **Panel A**: Illumina FASTQ sequenced read files are aligned to the human reference genome (hg19) using bowtie2, followed by removal of PCR duplicates, read group adjustment, InDel realignment, and base recalibration. Variant calling is conducted using the Unified Genotyper (parameters provided in **Materials and Methods**). Variant calling is conducted in parallel on all alignments. **Panel B**: The Affected Kindred/Cross-Cohort Filter makes use of known relatedness. Unshared variants between related samples are removed. Shared variants between unrelated samples are removed. Shared variants between related samples are retained. The Affected Kindred/Cross-Cohort Filter accommodates for the possibility that the absence of a variant in a related sample may also be due to insufficient coverage or variant quality in the related sample. All retained variants are subsequently run through the Shared Segment Filter.

The major component to the Affected Kindred/Cross-Cohort Filter involves a more sensitive implementation based on relatedness than the Shared Segment Filter. In this step, variants shared between two affected individuals from the same family are retained, while variants shared between two unrelated individuals in the small study cohort are removed. We constructed this filter to accommodate for variants that are discordant between affected kindred pairs where one variant is absent due to insufficient coverage, by simply retaining the variant with sufficient coverage. A schematic describing all steps from alignment to filtering using the Affected Kindred/Cross-Cohort filter is provided (Fig. 3).

Though the Affected Kindred/Cross-Cohort Filter is more sensitive than the Shared Segment Filter alone, it will not necessarily remove all the same passenger variants as the Shared Segment Filter. For example, shared common variants in the unshared chromosomal region of two affected kindred pairs will be retained by the Affected Kindred/Cross-Cohort Filter, because this filter is not aware if shared variants that are identical by state are also identical by descent. This step of the Affected Kindred/Cross-Cohort Filter results in the largest reduction (96.5%) in potentially neutral variants of all the individual filters (**Fig. 2, Row 6**).

Though the Affected Kindred/Cross-Cohort Filter relies solely on in-house data, it differs from other in-house filtering platforms that have been used previously. Importantly, this filter does not rely on using a separate negative control cohort of unaffected, unrelated individuals. Rather it relies on variant comparisons conducted within the same affected cohort, which is more akin to using an internal control. This has a number of unique advantages. Firstly, any reoccurring systematic errors that occur during library preparation or sequencing will self-neutralize. Secondly, more sequencing capacity can be devoted away from unaffected samples to affected samples, improving the power to detect disease-causing mutations. If at least some of this additional capacity is devoted to sequencing affected related sample pairs, that should further improves detection power. The main advantage of such a filter without additional controls is having greater detection power, which is one of the primary challenges when studying a disease with substantial locus heterogeneity.

### Comparison of Filters

Each filtering strategy above has its advantages and limitations. The advantage of dbSNP as a filter is that it is the largest annotated collection of variants available, and therefore removes the most variants from our cohort out of all the public database-derived filters (93.6% reduction; **Table 2, Row 5**). However, appropriate use of the database requires that it be modified to exclude as many known or possible pathological variants, or such variants risk being filtered out of the variant list of the study cohort. In this study, we removed known or probably pathological variants based on prior clinical annotation or MAF. Even so, this “Non-Clinical” dbSNP may still contain potentially functional variants relevant to our study cohort, and therefore this filter should be used cautiously.

Variants from males in the 1000 Genomes should serve as a valuable filter to remove neutral variants in our study. However, the publicly accessible dataset for males in the 1000 Genomes (*n* = 525) appears to contain ambiguous genotypes unique to variants from X-linked genes, and is therefore not suitable as an automatic filter to enrich for causal variants for rare X-linked disorders. To better assess whether this dataset can be used as a filter in our study, we generated variant calls directly from aligned sequence data for 162 males in the 1000 Genomes using the Unified Genotyper. The resulting variant dataset shows an excellent correlation with SNP genotype data (Omni Platform) made publically available through the 1000 Genomes Project. Filtering using this smaller variant dataset succeeded in removing 25.2% of potentially neutral variants (**Table 2, Row 3**). We expect that a similar dataset for all sequenced males in the 1000 Genomes project (*n* = 525) will likely achieve a greater reduction of neutral variants. Consistent with this expectation, filtering using the much larger EVS dataset (**Table 2, Row 4**) results in a 40.3% reduction in potentially neutral variants. We do not expect a full 1000 Genomes filter (males only) to outperform the “Non-Clinical” dbSNP filter, which consists of variants from both 1000 Genomes and alternate sources.

The Affected Kindred/Cross-Cohort Filter achieved the most substantial reduction in the average number of potentially neutral variants, demonstrating the robustness of this method (96.5% reduction; **Table 2, Row 6**). A large portion of the variants in dbSNP that have a MAF > 0.73% are re-discovered and removed given the sample size of our study, thus explaining why our filter outperforms the “Non-Clinical” dbSNP filter. Additionally, we have previously observed that false positive variant calls occur in parallel in multiple samples due to systematic errors in preparation or sequencing of the same library [22]. As an added advantage, our strategy removes these variants by comparing samples prepared in the same library, a process of self-neutralizing these reoccurring systematic errors.

Compared to the discrete filters based on public variant databases, our Affected Kindred/Cross-Cohort Filter shows superior performance at the removal of non-pathological variants in this XLID study. Importantly, this performance is achieved using a cohort size (*n* < 100) much smaller than those used in the 1000 Genomes, EVS, or aggregated into dbSNP. Given this performance, we predict that the sequencing of a large cohort of unrelated “normal” controls is not necessary. An additional advantage is that the Affected Kindred/Cross-Cohort Filter works independently from public reference databases and will therefore not be affected by any of the limitations noted previously for the database-dependent filters.

However, there is the disadvantage that, because this filter relies on the rare and private nature of XLID mutations, a potential causal mutation may be lost in the event that two affected but unrelated individuals possess that same mutation. This may be a result of incorrect ascertainment of relatedness, or because a disease-causing mutation by chance arose independently in two unrelated families. These events can be minimized by the confirmation of relatedness between samples and by rescuing recurrent causal mutations using dbSNP database (see below).

### Combination of Filters

**Fig 4:**
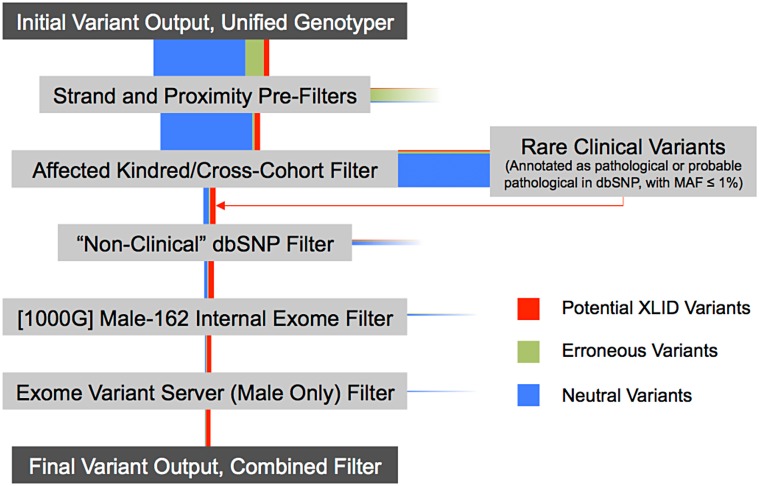
Schematic of Variant Reduction Using a Combined Filter. The Combined Filter sequentially applies all the filters described in this study. Vertical colored bars reflect relative changes in the content of the variant pool after each filter step. Horizontal colored bars reflect rejected variants upon each filter step. The Strand and Proximity Pre-Filters are applied universally. Then the Affected Kindred/Cross-Cohort Filter (with Shared Segment Filter) is applied. The rejected variant pool in this step primarily eliminates neutral variants. Nonetheless, this rejected pool of variants is assessed for co-occurrence with rare dbSNP variants with known pathological function. Rejected variants that positively co-occur in the Rare Clinical Variants dataset are re-introduced (thin red arrow). Database-dependent filters are sequentially applied. Red bars reflect potential XLID variants that may be of functional interest. Green bars reflect variants that are likely sequencing errors. Blue bars reflect variants that are likely neutral in XLID etiology.

Given these noted advantages and limitations, we tested one more filter that combines features from all of them. Firstly, the Shared Segment Filter and the Affected Kindred/Cross-Cohort Filter are applied sequentially. Secondly, we forcibly retain/re-introduce any variants lost in the Affected Kindred/Cross-Cohort Filter that are annotated as pathological in dbSNP and have a MAF < 1%. This retention would prevent the loss of known, rare disease-causing mutations that are shared between unrelated samples in the study cohort. Lastly, the three discrete public database-dependent filters (“Non-Clinical” dbSNP, [1000G] Male-162 Internal Exome, and Exome Variant Server) are used. Application of the database-dependent filters does not remove a significant number of neutral variants (already achieved in the first step), but it is nonetheless a simple series of filters to apply. A schematic of this combined filter is provided (Fig. 4).

The combination of these filters together results in 98.5% reduction in potentially neutral variants (**Table 2, Row 7**). Importantly, the number of variants for many samples is reduced to single-digit levels, thereby making downstream predictive and functional analyses much easier. Additionally, the re-introduction of pathological variants annotated in dbSNP succeeds in retaining a nonsense mutation, R37X, in ATRX. This mutation is a known cause for XLID, has been annotated as such in dbSNP, and occurs independently in our cohort in two unrelated individuals and one sibling pair. Because R37X in ATRX is shared between unrelated samples in our cohort, it was initially lost after application of the Affected Kindred/Cross-Cohort Filter. While such an event is rare due to broad allelic heterogeneity, retention of such a variant is preferable; these samples would otherwise be unnecessarily subjected to additional mutation discovery screens.

### Variant Validation by Sanger Sequencing

From the final variant list of the combined filter, we selected 19 potentially deleterious (17 coding and 2 splicing) variants and performed Sanger sequencing in 28 samples from the proband families. All 19 variants were positively confirmed in the respective 28 samples.

### Enrichment of Potential Causal Mutations

Our current enrichment system targets 975 genes on the X chromosome, of which 103 (10.6%) are known to cause XLID when mutated [1,27]. In order to determine if our combined filtering system could enrich for XLID genes, we sought to prioritize genes by mutation burden. The presence of at least one splicing or non-synonymous coding change in a sample would elevate the priority of a gene. Additional weight was given to nonsense and frame-shift mutations. No weight was given to intronic or synonymous mutations.

The resulting list consists of 89 X-linked genes (S1 File), of which 24 (27%) have been previously associated with XLID. Though kindred pairs are responsible for only 33.6% of the variants that were used to derive this list, they provided for a much greater enrichment (3.35-fold) of XLID genes compared to sporadic cases (1.39-fold). The expected fraction of XLID genes in the list should be 10.6%, not 27%. This 2.55-fold enrichment (kindred pairs and sporadic cases combined) for XLID genes over expected is statistically significant (*p* < 1E-5; hypergeometric test see **Materials and Methods**), demonstrating that our filtering system can be used quickly and effectively to re-identify known XLID genes. Given this enrichment, we hypothesize that the remaining portion of the list is also enriched for novel XLID genes. This approach has proven instrumental in the identification of novel XLID genes, including one gene that was prioritized at the top of our list, *ZC4H2*; this gene was recently implicated in Arthrogryposis Multiplex Congenita and Intellectual Disability [28]. A sample list of enriched genes and filtered variants is provided (Table 4).

**Table 4:** Identification of Known and Potentially Novel Genes for XLID Using X Chromosome Exome Sequencing and Affected Kindred/Cross-Cohort Analysis.

Name	Abbrev.	Map	Known or Predicted Function	XLID Gene	No. of Mutations	Mutation Nomenclature	SIFT Prediction	PolyPhen-2 Prediction	Variant Segregates with Disease in Family	Primary Isoform	Diseases or Phenotype	Reference
C4H2 domain-containing zinc finger	ZC4H2	Xq11.2	Zinc finger transcription factor		3	IVS+5G>A p.L66H p.R190W	NA Damaging Damaging	NA Prob. Damaging Prob. Damaging	Yes Yes Yes	NM_001178033	Wieacker-Wolfe Syndrome	Hirata et al, 2013
Alpha thalassemia / mental retardation syndrome X-linked	ATRX	Xq21.1	ATP-dependent helicase; chromosome remodeling	Yes	2	p.R37X p.S1606N	Known Damaging Damaging	Known Damaging Prob. Damaging	Yes Yes	NM_000489	XLID with alpha thalassemia	Gibbons et al, 1995
Ubiquitin-conjugating enzyme E2A	UBE2A	Xq24	Ubiquitin-conjugating enzyme	Yes	2	p.P68R p.Q110E	Damaging Tolerated	Prob. Damaging Prob. Damaging	Yes Yes	NM_003336	XLID, Nascimento-type	Nascimento et al, 2006
Filamin A	FLNA	Xq28	Actin-binding protein; cytoskeletal reorganization	Yes	2	p.G1576R p.F2228L	Damaging low conf Tolerated	Prob. Damaging Prob. Damaging	Yes	NM_001110556	Multiple congenital malformation syndromes	Fox et al, 1998
Transcription Initiation TFIID Subunit 1	TAF1	Xq13.1	Initiation of transcription by RNA Polymerase II and cell cycle control		2	p.M21L p.Q1428P	Tolerated Damaging	Benign Prob. Damaging	Yes	NM_138923		
Methly CpG-Binding Protein 2	MECP2	Xq28	Chromatin-based transcriptional regulation	Yes	1	p.K268E	Damaging	Prob. Damaging	Yes	NM_001110792	Rett Syndrome, XLID	Amir et al, 1999;; Schule et al, 2008; Orrico et al, 2000
Host cell factor C1	HCFC1	Xq28	Cell cycle control	Yes	1	p.G342S	Damaging	Prob. Damaging	Yes	NM_005334	XLID with methylmalonic acidemia	Huang et al, 2012; Yu et al, 2013
Zinc finger protein 711	ZNF711	Xq21.1	Zinc finger transcription factor	Yes	1	p.N601S	Damaging	Prob. Damaging	Yes	NM_021998	X-linked intellectual disability	Tarpey et al, 2009
Rho Guanine Nucleotide Exchange Factor 9	ARHGEF9	Xq11.1-q11.2	Brain-specific regulation of glycine and GABA receptors clusters	Yes	1	p.R236W	Damaging	Prob. Damaging	Yes	NM_001173480	XLID, Epileptic encephalopathy	Shimojima et al, 2011; Marco et al, 2008
E3 Ubiquitin Ligase	HUWE1	Xp11.22	Degradation of proteins involved in apoptosis and DNA maintenance	Yes	1	p.R4187H	Tolerated	Prob. Damaging	Yes	NM_031407	XLID, Turner-type	Turner et al, 1994
Ephrin B1	EFNB1	Xq13.1	Ligand of Eph-related receptor tyrosine kinases		1	p.G290R	Tolerated	Prob. Damaging	Yes	NM_004429	Craniofrontonasal Syndrome	Wieland et al, 2004; Twigg et al, 2004, 2013
Plexin A3	PLXNA3	Xq28	Semaphorin receptor; cytoskeletal remodeling		1	p.V1304M	Tolerated	Benign	TBD	NM_017514		
Ring finger protein 128	RNF128	Xq22.3	E3 Ubiquitin protein ligase		1	p.R12H	Damaging	Prob. Damaging	TBD	NM_194463		
Prickle homolog 3	PRICKLE3	Xp11.23	LIM domain-containing protein		1	p.R175C	Damaging	Prob. Damaging	Yes	NM_006150		
Zinc finger, RNA-binding motif and serine/arginine rich 2	ZRSR2	Xp22.2	Essential splicing factor		1	p.R440Q	Tolerated	Benign	Yes	NM_005089		
Glutamate receptor interacting protein associated protein 1	GRIPAP1	Xp11.23	Interaction with AMPA receptor complex		1	p.R822Q	Tolerated	Prob. Damaging	Yes	NM_020137		
O-linked N-acetylglucosamine Transferase	OGT	Xq13.1	Post-translational glycosylation		1	p.L244F	Tolerated	Prob. Damaging	Yes	NM_181673		
SRSF (Ser/Arg Splicing Factors) Protein Kinase 3	SRPK3	Xq28	Homolog of SRPK1 with possible role in splicing regulation		1	p.H159D	Damaging	Poss. Damaging	Yes	NM_014370		

## Discussion

We developed a strategy for rapid filtering of high-throughput sequencing data to identify disease-causing mutations in a rare X-linked Mendelian disorder with extensive locus and allelic heterogeneity. In sequencing affected samples with known relatedness in a small cohort of fewer than 100 samples, our Affected Kindred/Cross-Cohort Filter removes likely non-pathological variants to a level greater than that achieved using the largest publicly available variant dataset (dbSNP) as a filter. Variant sets from public databases or large control cohorts, though easily applied, are not required for effective filtration. This feature is important due to the intrinsic limitations of some of the public datasets. However, these public databases can be used if modified appropriately to compensate for the intrinsic limitations, as described above.

Using a combination of filters, we found a statistically significant enrichment for known XLID genes, strongly indicating that our method can be used to enrich for known disease-causing genes. Multiple novel candidate genes were also identified in this study, many of which are likely etiologic based on known biological function (Table 4 and S1 File).

*PLXNA3*, or Plexin A3, with Semaphorin, is involved in chemotactic signaling, a pathway involved in normal targeting of axonal projections in the central nervous system [29]. The Plexin-Semaphorin pathway has been previously implicated in an intellectual disability syndrome [30].

*GRIPAP1*, or *GRIP*-associated protein 1 (also known as *GRASP1*), is a neuronally expressed gene, with a role in glutamatergic AMPA receptor signaling, by regulating receptor trafficking and distribution through the Ras pathway [31]. *De* novo deleterious mutations in genes for glutamatergic signaling have been previously implicated in Intellectual Disability [32]. Additionally, glutamatergic receptors are dysregulated with loss of *FMR1* function, which is also a known cause of XLID [33,34]. *GRIPAP1* is located at Xp11, a region subject to duplication that has been previously associated with Autism with severe Intellectual Disability [35,36].

*EphrinB1* is ligand for Eph-related receptor tyrosine kinases and is involved in regulation of neuronal axon guidance [37]. Mutation of *EphrinB1* is a primary cause of Craniofrontonasal Syndrome, which can include symptoms of learning disability [38]. An *EphrinB1* deficient mouse model of Craniofrontonasal Syndrome shows cortical abnormalities and learning deficits [39].

*OGT*, or O-linked N-acetylglucosamine Transferase, is an essential factor that catalyzes the post-translation modification or serine and threonine residues. Additionally, *OGT* forms a complex with TET proteins in the nucleus to regulate chromatin [40]. This complex includes *HCFC1*, a target of *OGT*, which is another gene implicated in XLID [41].

Our results demonstrate a robust reduction in variants and a significant enrichment for known and putative disease causing genes. In complementation with other mutation prioritization options like functional variant prediction analysis, the Affected Kindred/Cross-Cohort Filter can identify causal mutations in rare, heterogeneous X-linked disorders such as XLID.

## Materials and Methods

### Study Sample

Genomic DNA samples from males with XLID (*n* = 82) were sequenced. Of the 82 samples, 30 are sporadic cases and are unrelated to other individuals in the study cohort. The remaining 52 samples constitute 26 kindred pairs, with relationships described in Table 1. X-linkage was determined by at least one of four criteria: 1) the responsible locus was mapped by linkage analysis or a similar method to the X chromosome; 2) affected males in two or more generations in their pedigree show a pattern of inheritance consistent with X-linkage; 3) two or more affected males in the same generation in their pedigree are consistent with X-linked inheritance, with evidence for a skewed (90:10) pattern of X-inactivation in females; 4) the presentation of disease is consistent with the clinical diagnosis of a known, well-defined XLID syndrome for which the causative gene is unknown. All the samples were previously tested as negative for Fragile X Syndrome, cytogenetic abnormalities, and known inborn errors of metabolism. An informed consent was obtained from all families enrolled in this study at the Greenwood Genetic Center, SC, and/or the Johns Hopkins University. The Institutional Review Board from the respective institutions approved this study.

### Library Preparation and Sequencing

Sequencing libraries were prepared using the TruSeq^TM^ DNA Sample Preparation kit following a standard protocol from the manufacturer (Illumina). Twelve or 24 individually indexed libraries were pooled at equal molar ratio and enriched for the X-exome using a SureSelect Human X Chromosome Exome Kit (Agilent). Each pooled library was quantified by qPCR using a KAPA library quantification kit (KAPA Biosystems) and sequenced in one lane of HiSeq2000 using 75bp pair-end sequence module.

### Sequence Data Analysis

Bowtie2 was used to align fastq reads using the [—very-sensitive-local] parameter and other Bowtie2 parameters were kept at default [42]. PCR duplicates were removed using Picard (http://picard.sourceforge.net). Indel realignment and base recalibration was conducted using GATK [43]. Unified Genotyper (GATK) was used for variant calling with the ploidy parameter setting at “1” (haploid) due to the hemizygous nature of the male X chromosome [44]. The pseudoautosomal regions of the X chromosome were not included in our study. Additional pre-filtering parameters were instituted based on strand-specific coverage and variant proximity to reduce the false positive rate in variant calling [22]. By comparing variant calls from the Unified Genotyper to nucleotide pileups from the aligned reads, this filter only retains variants with at least one alternate base call from each strand and only retains variants that are not present within ten nucleotides of another variant. This filter is applied in addition to the default *FisherStrand* covariate analysis conducted during base recalibration by GATK. Variants were annotated for affected genes and coding changes using the ANNOVAR package [45].

### Estimation of the Relatedness between Samples

The relatedness between samples was determined based on the fraction of total variant calls that are identical between each sample in the entire cohort [Identity ≈ (2 x number of variants identical between both samples)/(sum of variants of both samples)]. The closely related samples such as affected brothers and maternal cousins from the sample families generally shared the greatest identity, which confirms their expected relatedness (Fig. 1). Relatedness was also validated visually using the Shared Segment filter step of the Affect Kindred/Cross-Cohort Filter (S1 Fig.).

### Identification of Shared Segment that Are Identical By Descent

Identification of regions of sharing between related samples was conducted using an automated 5 MB sliding window across all possible section of IBD. The regions defined by the sliding window were then manually refined. To determine if shared IBD segments align along known linkage intervals, SNPs that are in linkage were assessed for crossover along the boundaries of the shared segments. Retrieval of linked SNPs was conducted using PLINK v1.07 [plink—bfile hapmap3_r2_b36_fwd.consensus.qc.poly—blocks—noweb—chr X—from-bp 1—to-bp 155270560—missing-phenotype 1] on HapMap Phase 3 data (MAP and PED files) [46,47].

### Determination of Population Stratification

Population stratification was determined by comparing samples to a cohort mean, and by comparing sample allele frequencies to population-specific allele frequencies derived from EVS data. For the cohort mean analysis, an average allele frequency for the cohort was obtained for each variant. A residual sum of squares was subsequently calculated for each sample compared to the cohort mean. The number of standard deviations from the residual is plotted on the Y-axis of S3a Fig. Comparison of sample allele frequencies to EVS was performed for both EVS European American (EA) and EVS African American (AA) population frequencies. For any given sample, an EA and an AA metric was obtained by averaging the population specific allele frequencies for all sample variants present in the EVS dataset. This EA metric for each sample is plotted on the X-axis of S3 Fig. The AA metric for each sample is plotted on the Y-axis of S3b Fig.

### Hypergeometric Test for Known XLID Gene Enrichment

The full X chromosome gene set targeted by exome sequencing includes 975 coding genes, of which 103 are attributed to XLID in literature. Our combined filtering pipeline resulted in a large reduction in likely non-causal variants. When these variants are scored by function (nonsense, splicing, missense), we obtained a list of 89 prioritized genes, of which 24 are attributed to XLID in literature. A hypergeometric test was used to determined the probability of obtaining at least 24 (*k*) known XLID genes with 89 (*n*) random draws from a population of 975 (*N*) genes, of which only 103 (*K*) are known XLID genes. The p-value for an over-representation of known XLID genes is the probability of randomly drawing *k* or more XLID genes with a total of *n* genes drawn from the total population of *K* in *N*, without replacement. (R code: [sum(dhyper(*k*:*n*, *K*, *N*-*K*, *n*))] or [1-phyper(*k*-1, *K*, *N*-*K*, *n*)]). This p-value was calculated to be less than 4E-6. As a negative control, we calculated the mutation frequency for each X chromosome gene, using our 162 male 1000G negative control cohort. It is necessary to take into consideration the mutation rate for each gene, as some XLID genes, such as DMD and MECP2 have higher mutation rates, which will increase the likelihood of enrichment. The probability of randomly selecting an XLID gene from the total X chromosome gene set, weighted for genic mutation rate, was calculated to be 0.103408.

### Validation of Variants by Sanger Sequencing

Sanger sequencing for variant validation was conducted using the BigDye Terminator v3.1 Cycle Sequencing Kit on an ABI3100 automatic DNA analyzer (Applied Biosystems) following manufacturer’s instructions. Analysis was done on standard sequence alignment software (CodonCode and MacVector) followed by manual investigations of the chromatograms.

### “Non-Clinical” dbSNP, Male 1000 Genomes, and Male EVS as Variant Filters

A “Non-Clinical” dbSNP dataset was generated by the removal of variants that are present in the clinvar databases (CLINSIG = 4 [probable-pathogenic] or 5 [pathogenic]) from dbSNP (build 137), followed by the removal of variants with MAF < 0.73%. For the 1000 Genomes project, variant data (SNPs and INDELs) from male samples only (*n* = 525) were extracted from the master variant output of the 1000 Genomes project (Integrated Phase 1, version 3: 20101123). SNP genotyping data (Omni Platform) was used to correlate variants calls from males in the 1000 Genomes. An additional variant call list, using default Unified Genotyper parameters, was conducted on 162 males samples from the 1000 Genomes alignment files. This alternate variant list was generously provided by the Chakravarti Lab (Johns Hopkins University). For the Exome Variant Server dataset, variants present in the male portion of the non-pseudoautosomal regions of the X chromosome were extracted based on variant annotations of hemizygous genotype.

### Analysis Pipelines

Computational scripts for data analysis in this study are provided (S2 File).

## Supporting Information

S1 Fig Shared IBD Segments for All Samples. Plot titles indicate sample identifiers (Sample Pair 1—Sample Pair 2). X-axis denotes position along the X chromosome. Far left is position 1 and far right is position 154,899,846, relative to the hg19 reference sequence. Black vertical bars indicate positions along the X chromosome at which a variant was called in one sample, but was not called in its paired sample (genotypic discordance between related samples). Orange blocks reflect regions lacking an abundance of discordant genotypes. These regions are shared Identical by Descent (inheritance) between the samples and contain the pathological variants of interest. All variants, both genotypically concordant (not shown) and discordant, that are located within the orange blocks are retained by the Shared Segment Filter. All possible pairwise relationships were assessed for segment sharing by an automated 5 MB sliding window and manual curation. Only sample with substantial sharing are plotted.
(PDF)

S2 Fig Correlation Between Shared Segments of IBD and Known Linkage Intervals. Plot titles indicate sample identifiers (Sample Pair 1—Sample Pair 2). X-axis denotes position along the X chromosome. Far left is position 1 and far right is position 154,899,846 relative to the hg19 reference sequence. Orange blocks reflect regions determined to be in IBD. These regions are shared by inheritance between the samples and contain the pathological variant of interest. Black dots are HapMap SNPs and are distributed across the Y-axis based on genotypic sharing between the sample pairs. SNP positions retaining the reference allele between both samples (Concordant Ref) are located at the bottom in the pink region. SNP positions retaining the alternate allele between both samples (Concordant Alt) are located at the middle in the green region. SNP positions that are genotypically discordant between the sample pairs (Discordant) are located at the top in the blue region. Individual black dots reflect one SNP position. Vertical black lines connecting dots reflect SNPs that are known to be linked as determined using PLINK on HapMap Phase 3 data. Vertical lines rarely cross the orange IBD shared segments, suggesting that these segments are likely descending along known linkage intervals. However, only higher resolution, full chromosome genotyping (not exome sequence) can prove this conclusively.
(PDF)

S3 Fig Assessment of Cohort Population Stratification. Population stratification was determined by comparing samples to a cohort mean, and by comparing sample allele frequencies to population-specific allele frequencies derived from EVS data. For the cohort mean analysis, an average allele frequency for the cohort was obtained for each variant. A residual sum of squares was subsequently calculated for each sample compared to the cohort mean. The number of standard deviations from the residual is plotted on the Y-axis of S3a Fig.. Comparison of sample allele frequencies to EVS was performed for both EVS European American (EA) and EVS African American (AA) population frequencies. For any given sample, an EA and an AA metric was obtained by averaging the population specific allele frequencies for all sample variants present in the EVS dataset. This EA metric for each sample is plotted on the X-axis of S3 Fig. The AA metric for each sample is plotted on the Y-axis of S3b Fig. The majority of samples cluster together with genotypes of primarily European ancestry. However, five samples show slight deviations from the main cluster. When SNP loads were compared to EVS data from the African American population, the sample cluster deviation was reproduced, indicating that these five samples have a small, but detectable contribution of African ancestry.
(PDF)

S1 Table Calculation of Rare Minor Allele Frequency (MAF) Cutoff. S1 Table describes the probability of observing *n* number of mutant alleles in the male-only sub-population of the 1000 Genomes project without observation of the mutant allele at all in the female sub-population. S1 Table shows that the probability of observing 12 or more copies of the mutant allele in males only is less than 1%, which commends this allele count as an appropriate cutoff for the minor allele frequency.
(PDF)

S1 File List of 89 Potential XLID Genes. Listed are 89 genes that were found mutated in our XLID cohort after application of the Combined Filter. Genes were prioritized by presence of a missense, nonsense, or splicing mutation. Weight was given for nonsense and splicing mutations. Weight was given for mutations present in both siblings. An asterisk (*) after the Gene ID indicates that the gene has been previously associated with XLID. The current list of XLID genes as of this publication stands at 103 (see bottom of list). Our target-enrichment system targets 975 chromosome X genes. Of the 89 genes in the list, 24 have been previously associated to XLID. This reflects a statistically significant 2.55-fold enrichment for known or previously associated XLID genes (p < 1E-5, hypergeometric test).
(TXT)

S2 File Scripts and Algorithms utilized for XLID Study. S2 File is a compressed file providing a collection of bash-based and R-based scripts that were written and run to process and analyze our XLID study cohort. The scripts provided assume that short-read alignment has already been performed, including quality correction steps. These scripts will run data processing and analysis for a test cohort, as well as comparison analyses with other public databases.
(ZIP)
