# Supplementary material for: Affected Kindred Analysis of Human X Chromosome Exomes to Identify Novel X-Linked Intellectual Disability Genes
Source: PLoS One. 2015 Feb 13;10(2):e0116454. doi: 10.1371/journal.pone.0116454 (PMC4332666; doi:10.1371/journal.pone.0116454)
Supplement: S1 Fig — Plot titles indicate sample identifiers (Sample Pair 1—Sample Pair 2). X-axis denotes position along the X chromosome. Far left is position 1 and far right is position 154,899,846, relative to the hg19 reference sequence. Black vertical bars indicate positions along the X chromosome at which a variant was called in one sample, but was not called in its paired sample (genotypic discordance between related samples). Orange blocks reflect regions lacking an abundance of discordant genotypes. These regions are shared Identical by Descent (inheritance) between the samples and contain the pathological variants of interest. All variants, both genotypically concordant (not shown) and discordant, that are located within the orange blocks are retained by the Shared Segment Filter. All possible pairwise relationships were assessed for segment sharing by an automated 5 MB sliding window and manual curation. Only sample with substantial sharing are plotted. (PDF) [file pone.0116454.s001.pdf]

CMS14284 – CMS15703–K9427

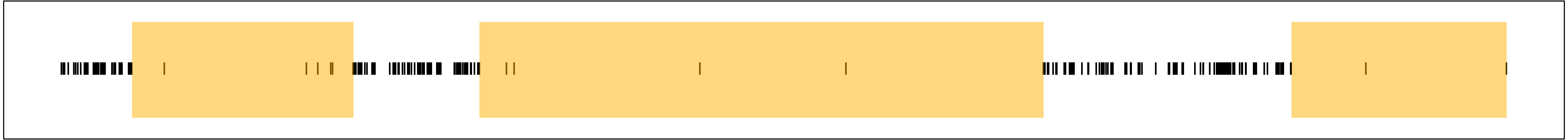

X Chromosome: Pos 1 – 154,899,846

4965 – 4966

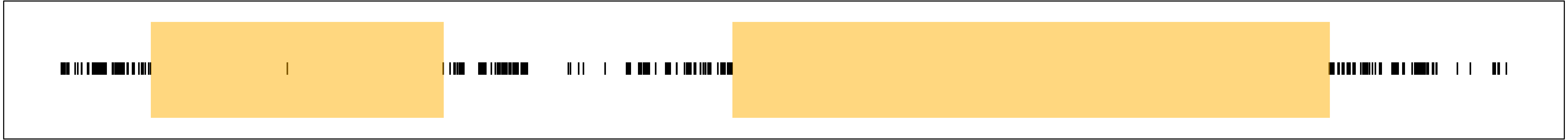

X Chromosome: Pos 1 – 154,899,846

5896 – 5897

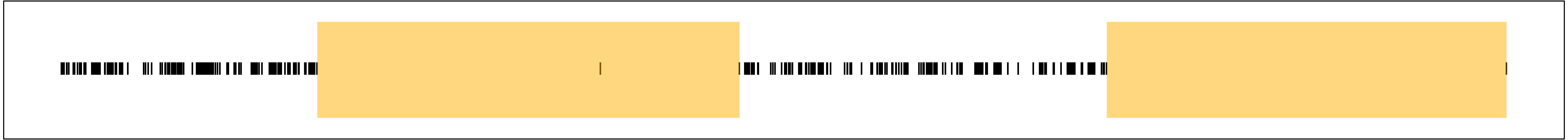

X Chromosome: Pos 1 – 154,899,846

cms0245 – cms0564

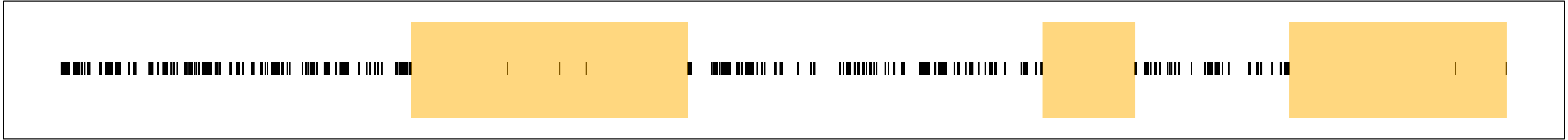

X Chromosome: Pos 1 – 154,899,846

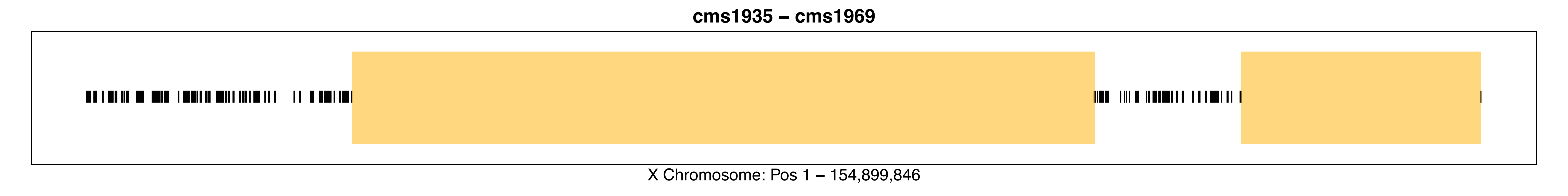
cms1935 – cms1969
X Chromosome: Pos 1 – 154,899,846

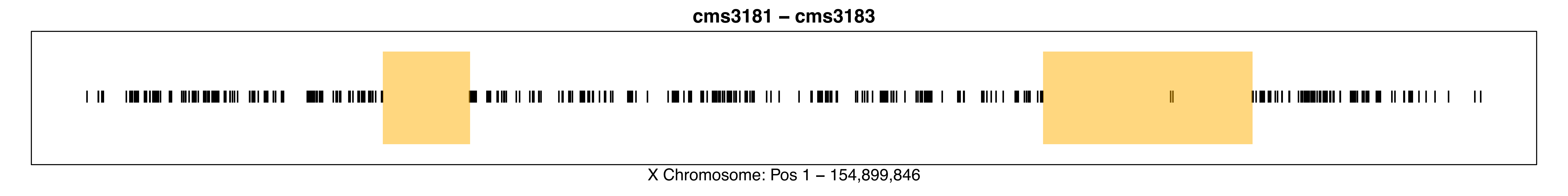
cms3181 – cms3183
X Chromosome: Pos 1 – 154,899,846

cms4418 – cms4419

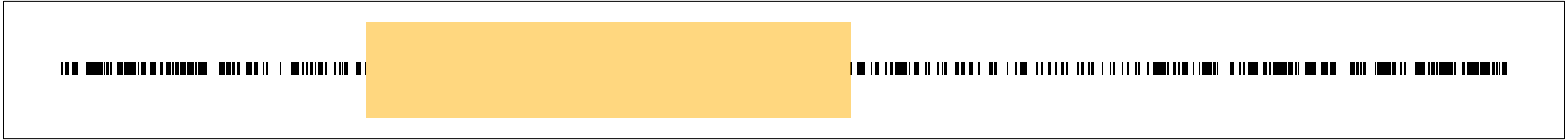

X Chromosome: Pos 1 – 154,899,846

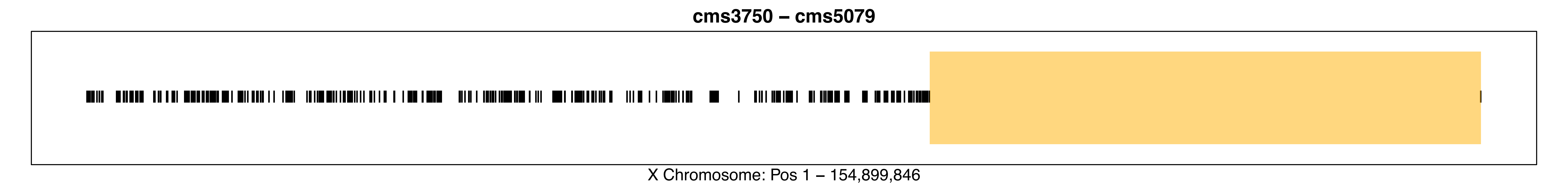
cms3750 – cms5079
X Chromosome: Pos 1 – 154,899,846

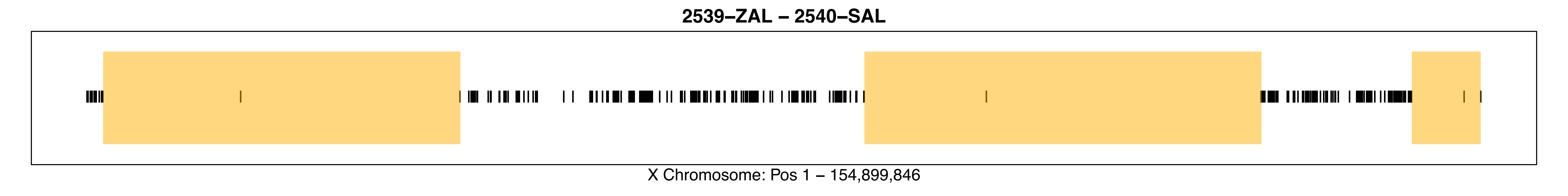
2539–ZAL – 2540–SAL
X Chromosome: Pos 1 – 154,899,846

Griffin–J – Griffin–R

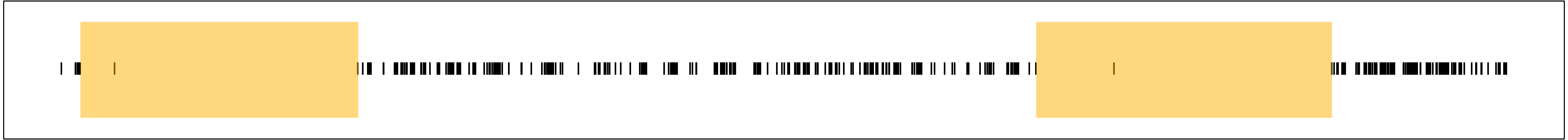

X Chromosome: Pos 1 – 154,899,846

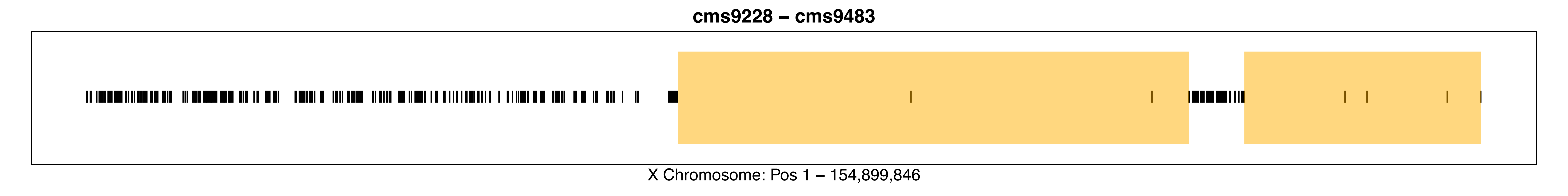
cms9228 – cms9483
X Chromosome: Pos 1 – 154,899,846

cms11961 – cms11962

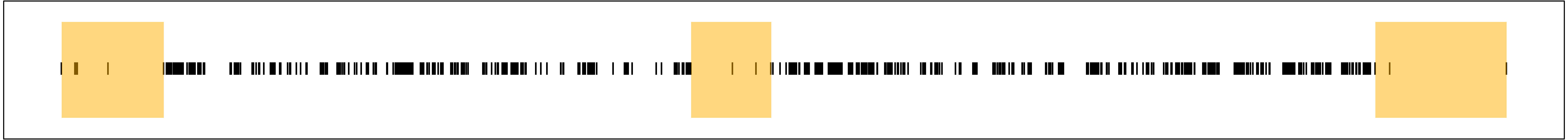

X Chromosome: Pos 1 – 154,899,846

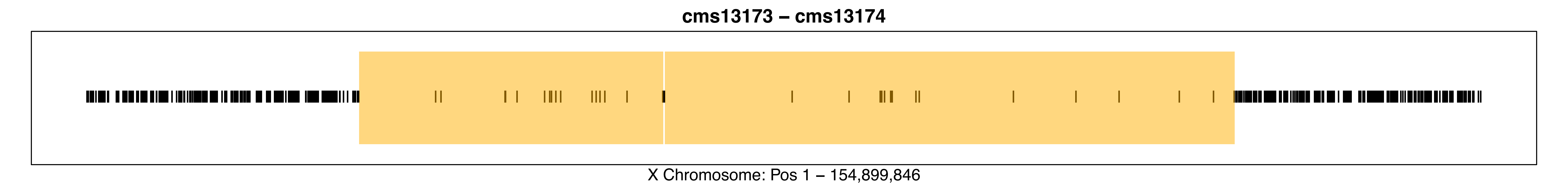
cms13173 – cms13174
X Chromosome: Pos 1 – 154,899,846

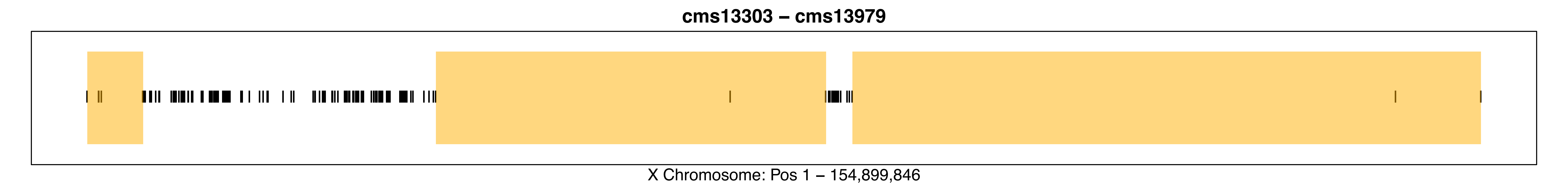
cms13303 – cms13979
X Chromosome: Pos 1 – 154,899,846

cms17118 – cms18212

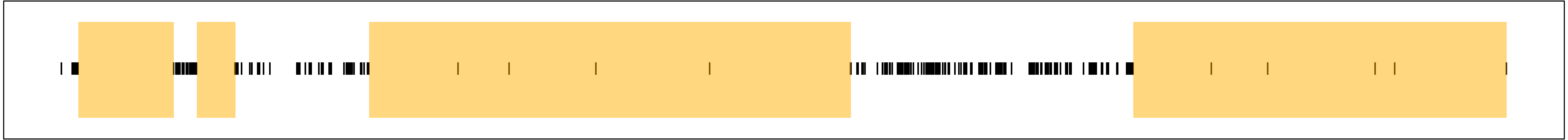

X Chromosome: Pos 1 – 154,899,846

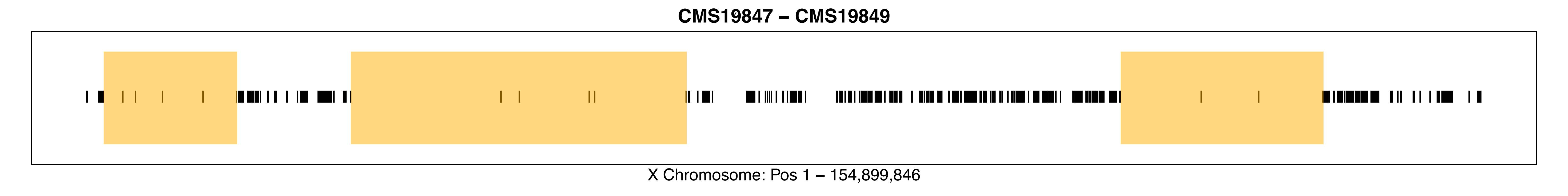
CMS19847 – CMS19849
X Chromosome: Pos 1 – 154,899,846

cms22744 – cms22745

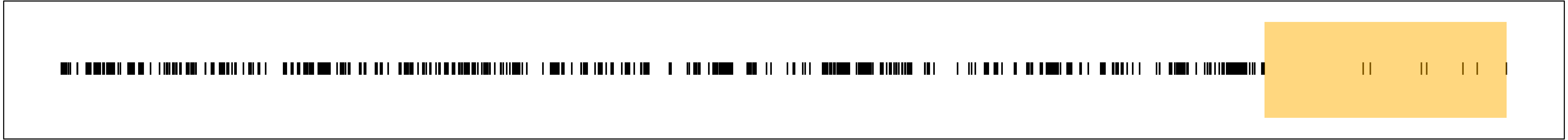

X Chromosome: Pos 1 – 154,899,846

cms13265 – cms13267

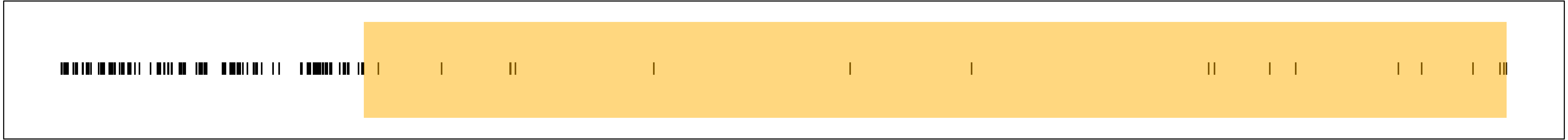

X Chromosome: Pos 1 – 154,899,846

13439 – cms1861

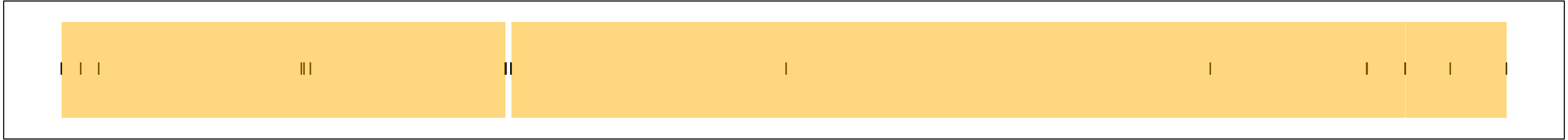

X Chromosome: Pos 1 – 154,899,846

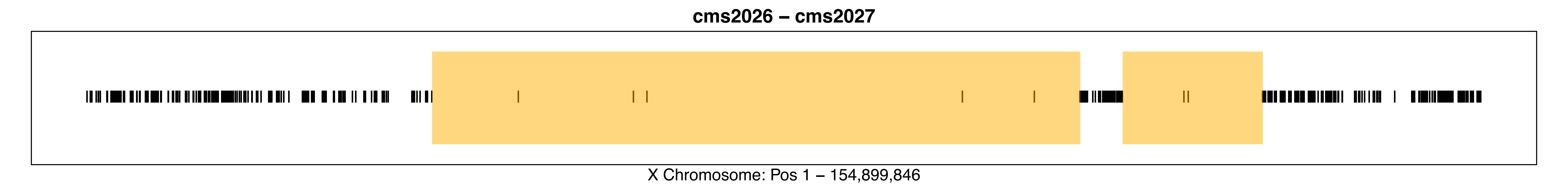
cms2026 – cms2027
X Chromosome: Pos 1 – 154,899,846

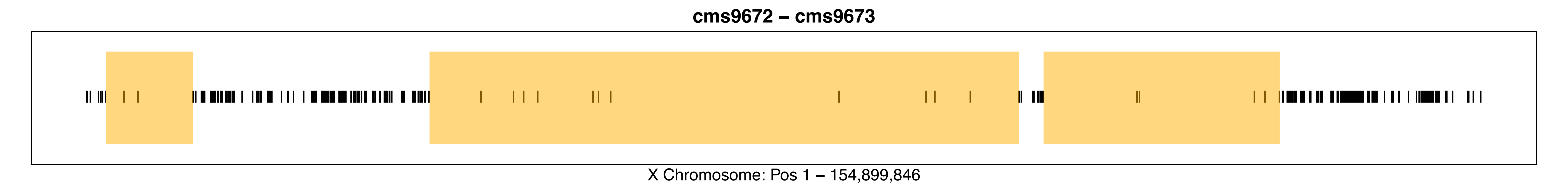
cms9672 – cms9673
X Chromosome: Pos 1 – 154,899,846

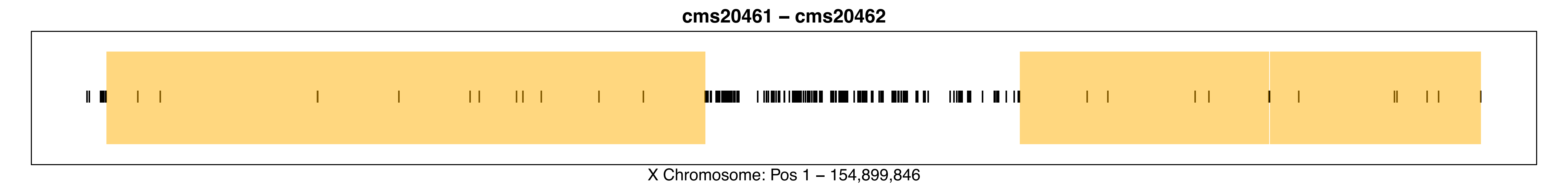

cms18655 – cms18656

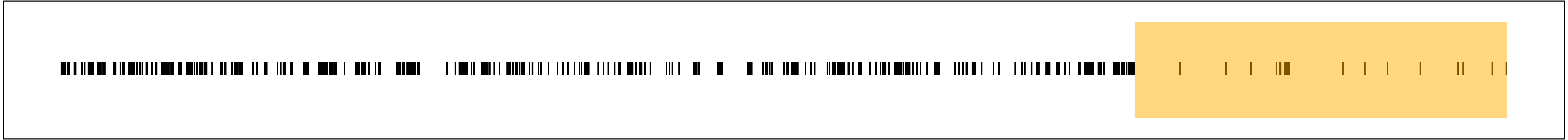

X Chromosome: Pos 1 – 154,899,846

CMS20461 – CMS20462

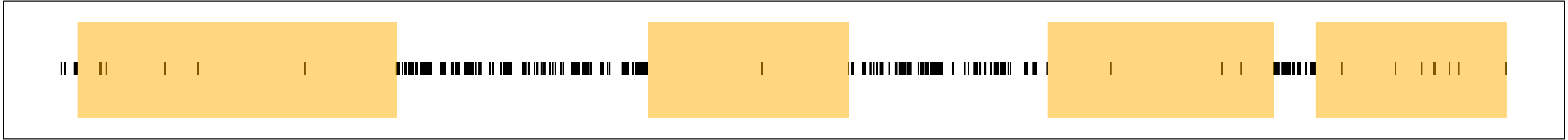

X Chromosome: Pos 1 – 154,899,846

cms0559a – cms19029

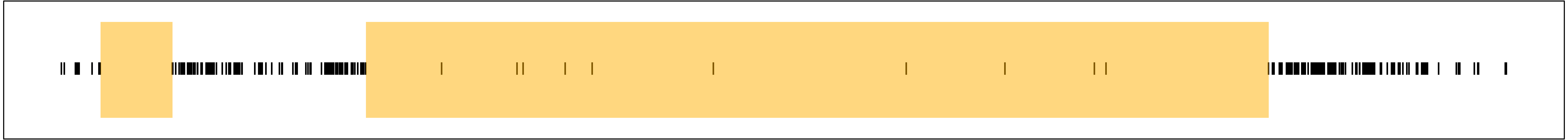

X Chromosome: Pos 1 – 154,899,846

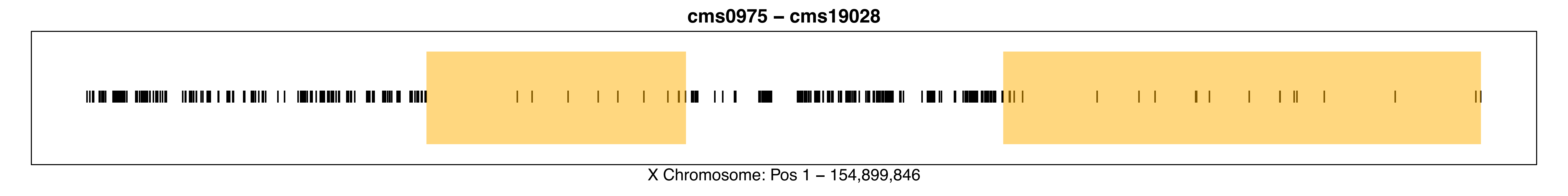
cms0975 – cms19028
X Chromosome: Pos 1 – 154,899,846
