# Supplementary material for: Affected Kindred Analysis of Human X Chromosome Exomes to Identify Novel X-Linked Intellectual Disability Genes
Source: PLoS One. 2015 Feb 13;10(2):e0116454. doi: 10.1371/journal.pone.0116454 (PMC4332666; doi:10.1371/journal.pone.0116454)
Supplement: S2 Fig — Plot titles indicate sample identifiers (Sample Pair 1—Sample Pair 2). X-axis denotes position along the X chromosome. Far left is position 1 and far right is position 154,899,846 relative to the hg19 reference sequence. Orange blocks reflect regions determined to be in IBD. These regions are shared by inheritance between the samples and contain the pathological variant of interest. Black dots are HapMap SNPs and are distributed across the Y-axis based on genotypic sharing between the sample pairs. SNP positions retaining the reference allele between both samples (Concordant Ref) are located at the bottom in the pink region. SNP positions retaining the alternate allele between both samples (Concordant Alt) are located at the middle in the green region. SNP positions that are genotypically discordant between the sample pairs (Discordant) are located at the top in the blue region. Individual black dots reflect one SNP position. Vertical black lines connecting dots reflect SNPs that are known to be linked as determined using PLINK on HapMap Phase 3 data. Vertical lines rarely cross the orange IBD shared segments, suggesting that these segments are likely descending along known linkage intervals. However, only higher resolution, full chromosome genotyping (not exome sequence) can prove this conclusively. (PDF) [file pone.0116454.s002.pdf]

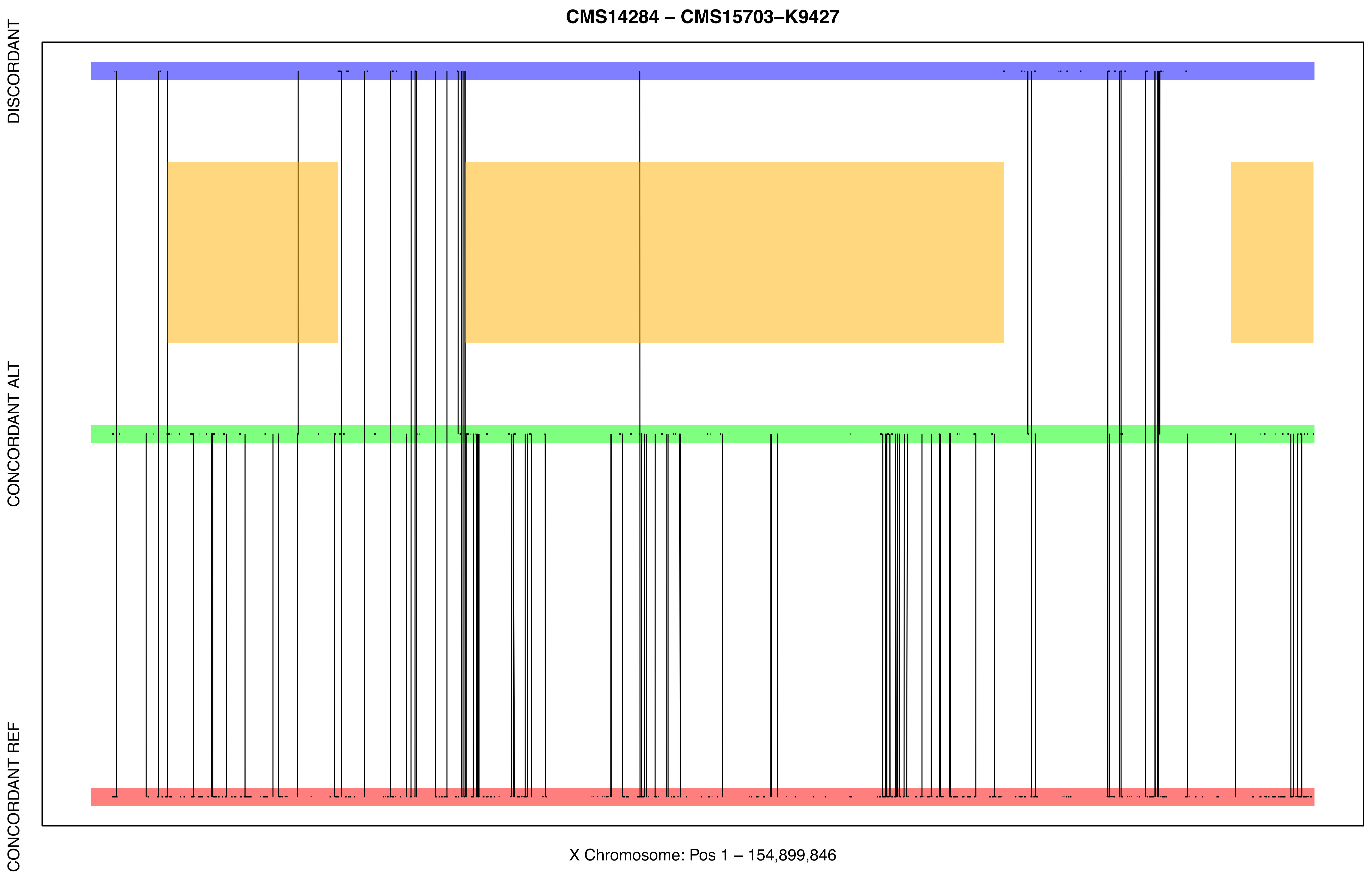

CMS14284 – CMS15703–K9427
DISCORDANT
CONCORDANT ALT
CONCORDANT REF
X Chromosome: Pos 1 – 154,899,846

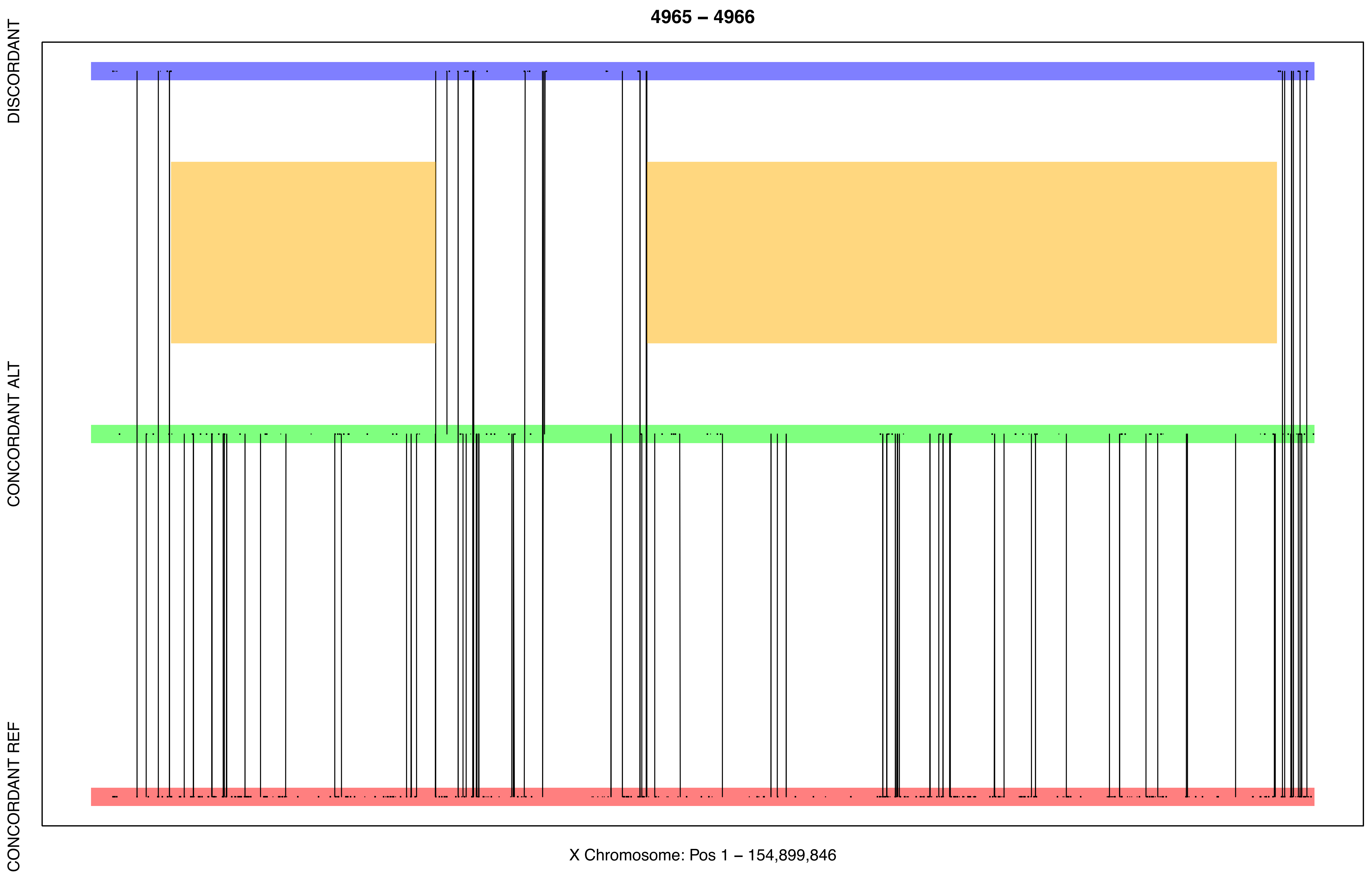

4965 – 4966
DISCORDANT
CONCORDANT ALT
CONCORDANT REF
X Chromosome: Pos 1 – 154,899,846

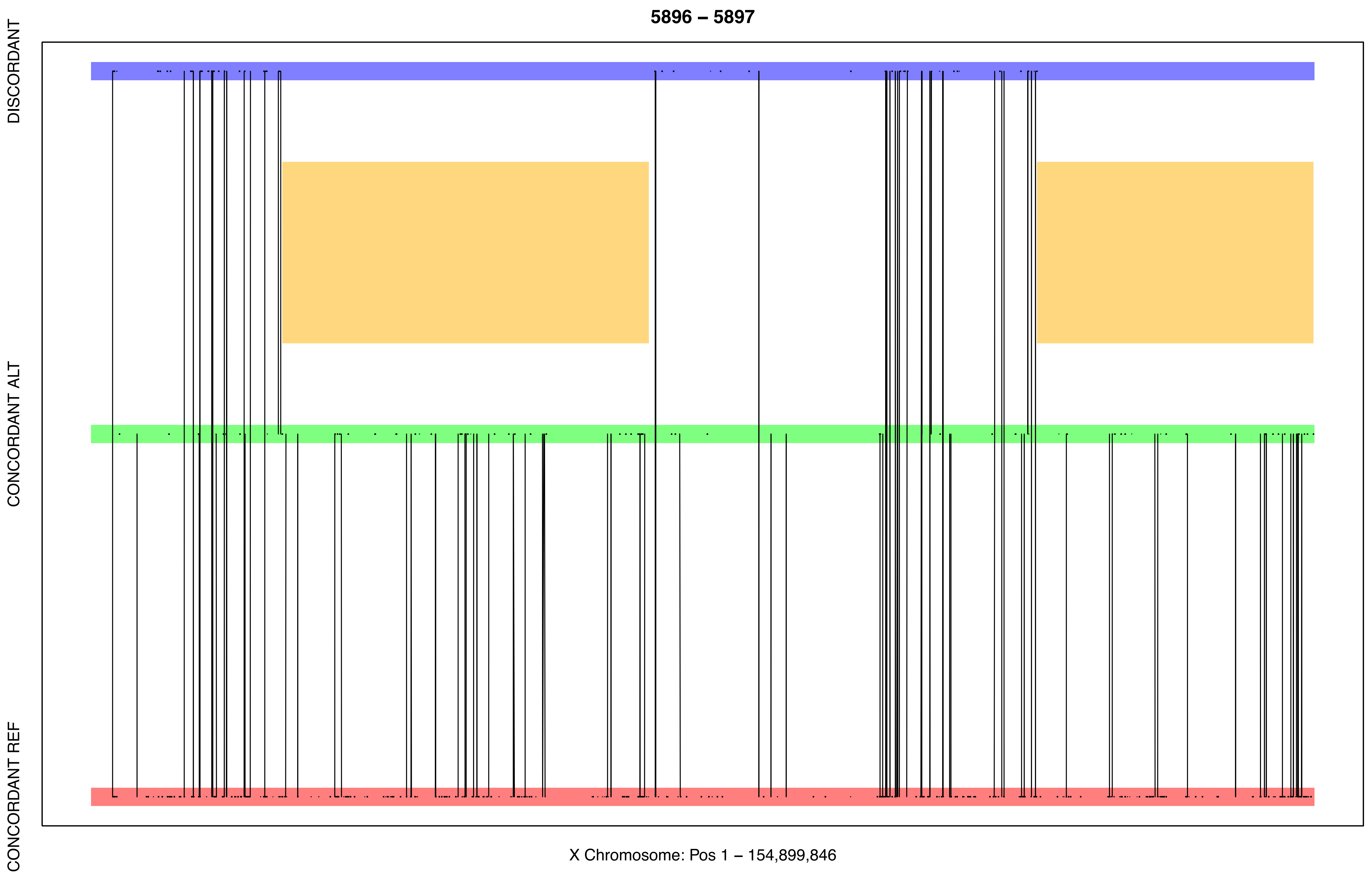
5896 – 5897
DISCORDANT
CONCORDANT ALT
CONCORDANT REF
X Chromosome: Pos 1 – 154,899,846

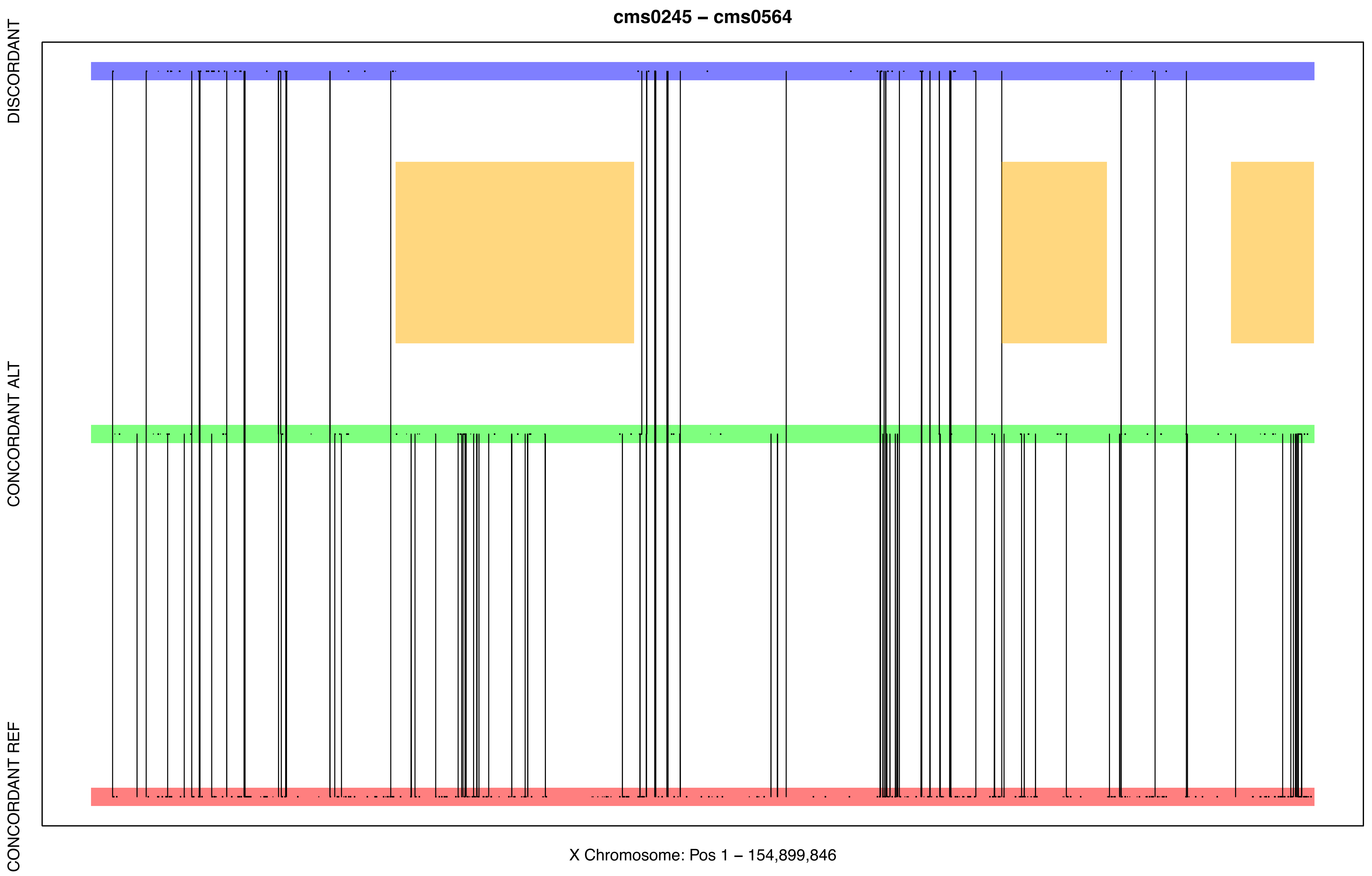
cms0245 – cms0564
DISCORDANT
CONCORDANT ALT
CONCORDANT REF
X Chromosome: Pos 1 – 154,899,846

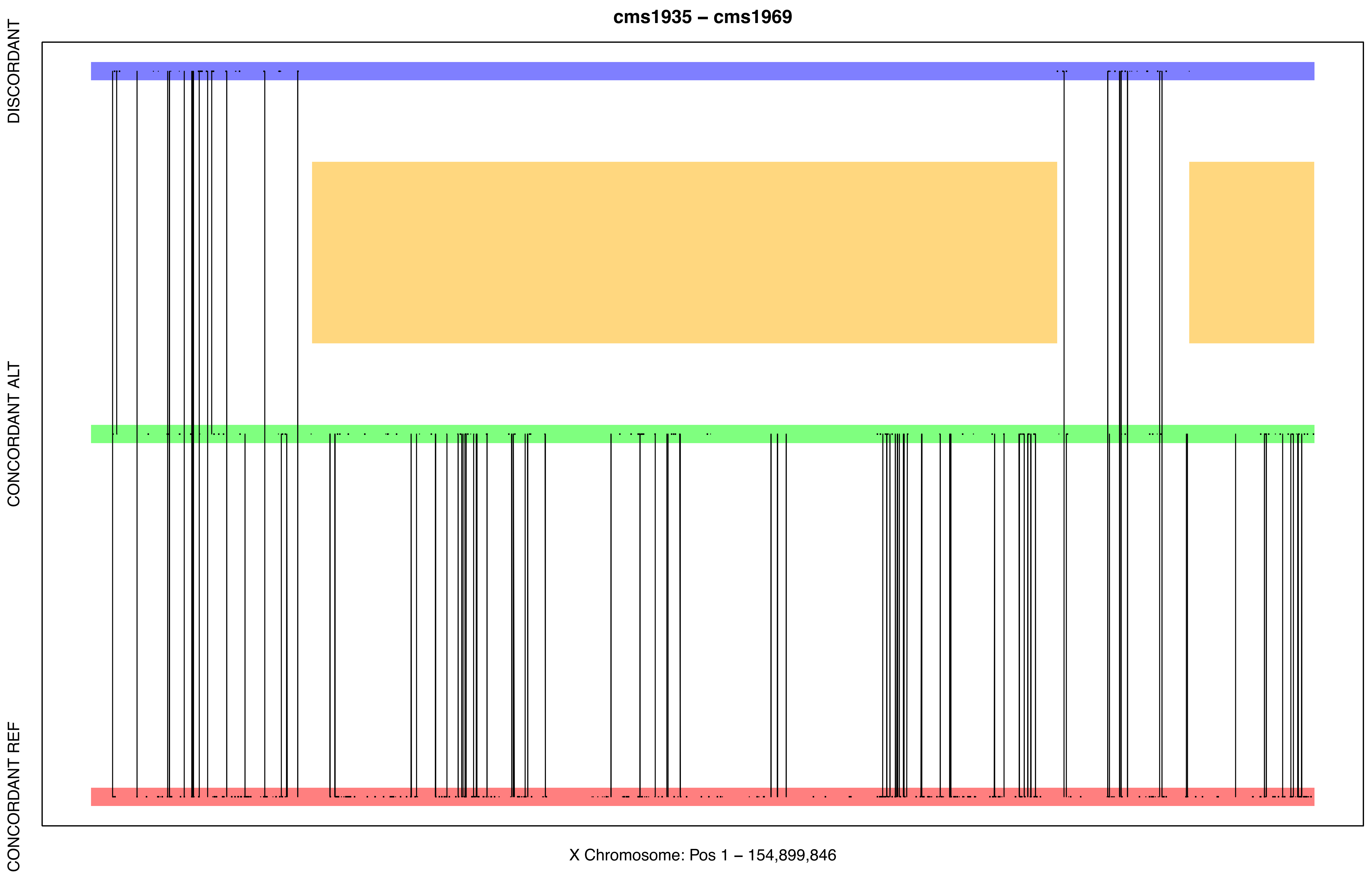
cms1935 – cms1969
DISCORDANT
CONCORDANT ALT
CONCORDANT REF
X Chromosome: Pos 1 – 154,899,846

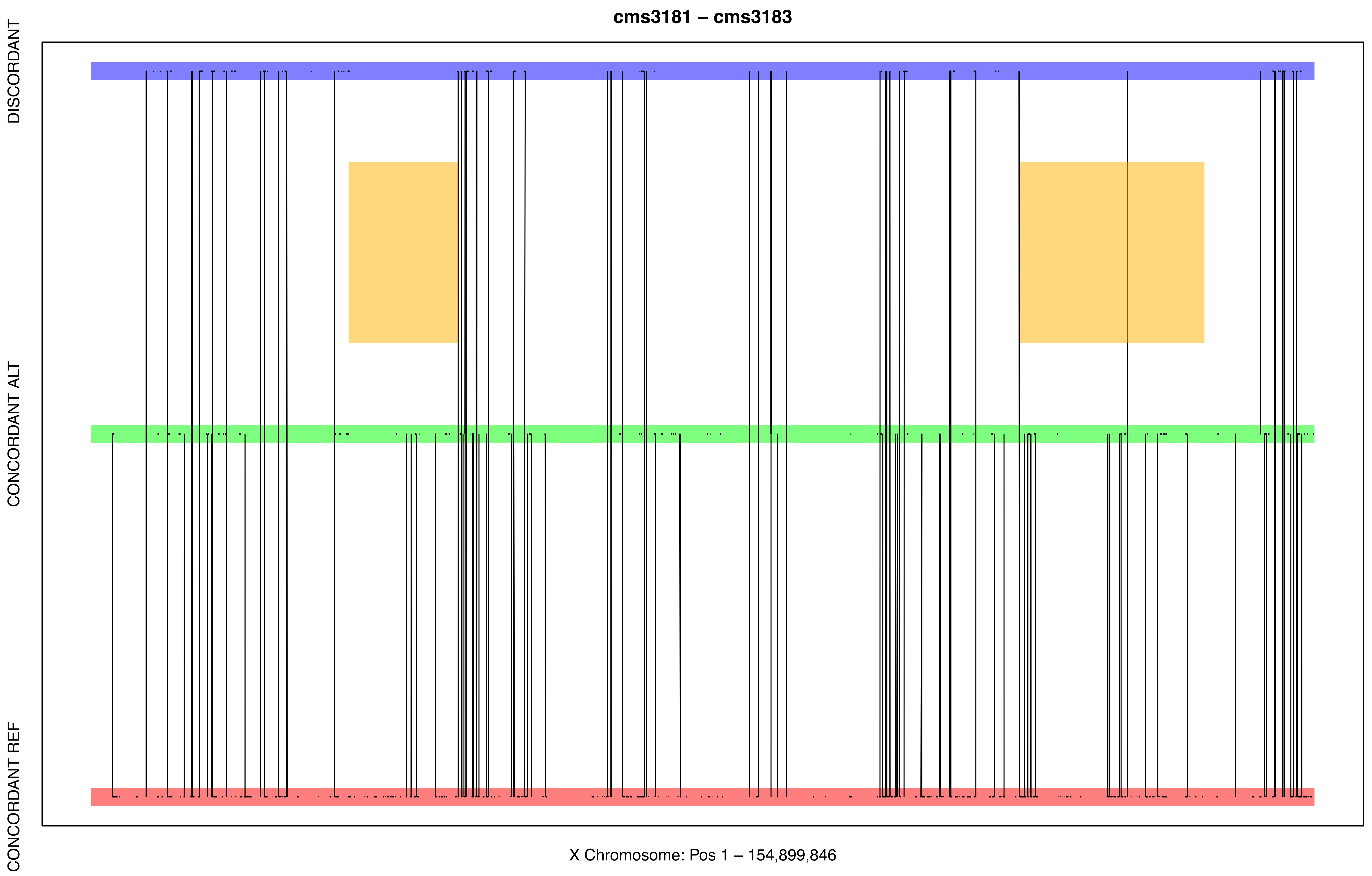

cms3181 – cms3183
DISCORDANT
CONCORDANT ALT
CONCORDANT REF
X Chromosome: Pos 1 – 154,899,846

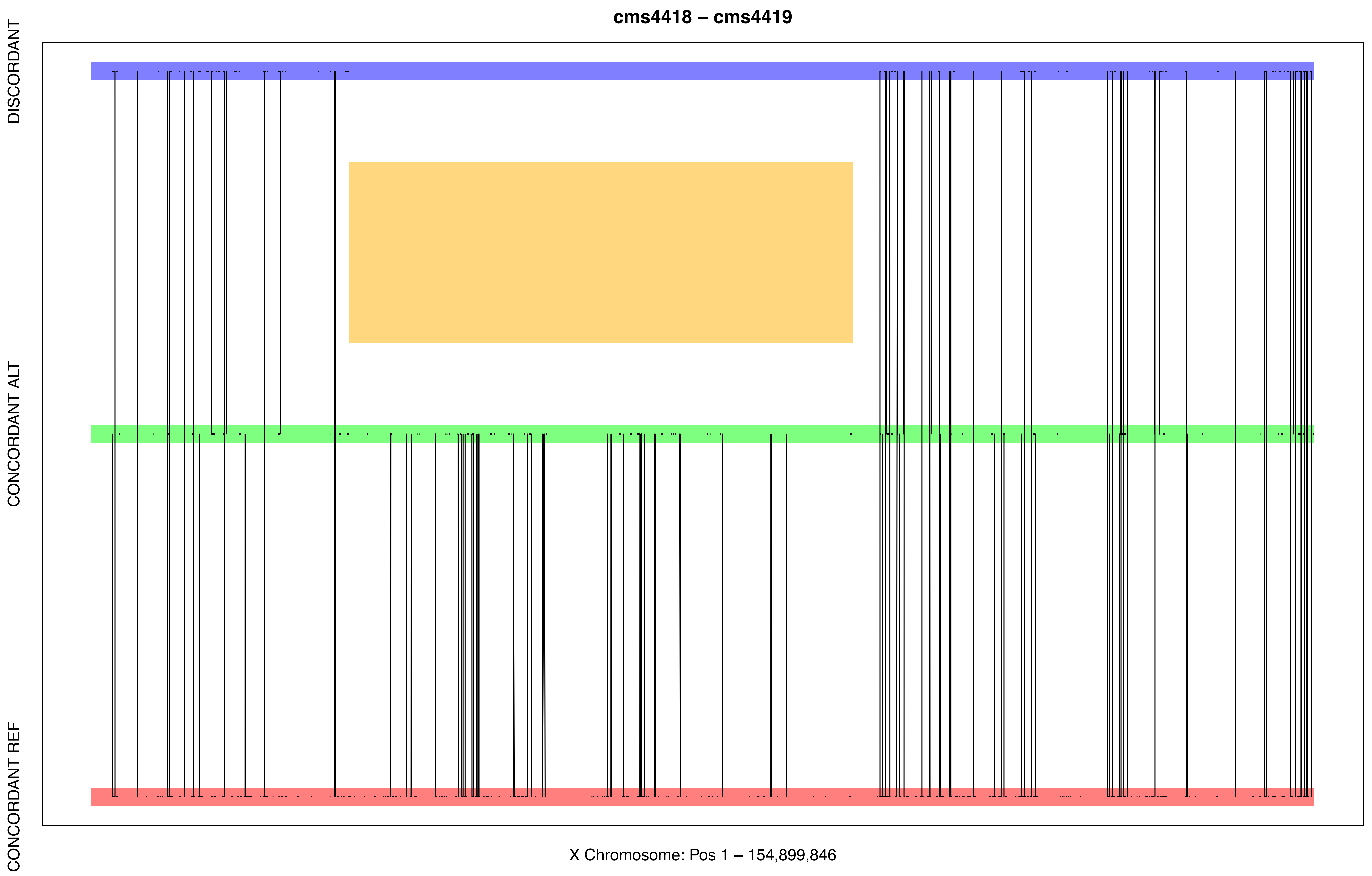
cms4418 – cms4419
DISCORDANT
CONCORDANT ALT
CONCORDANT REF
X Chromosome: Pos 1 – 154,899,846

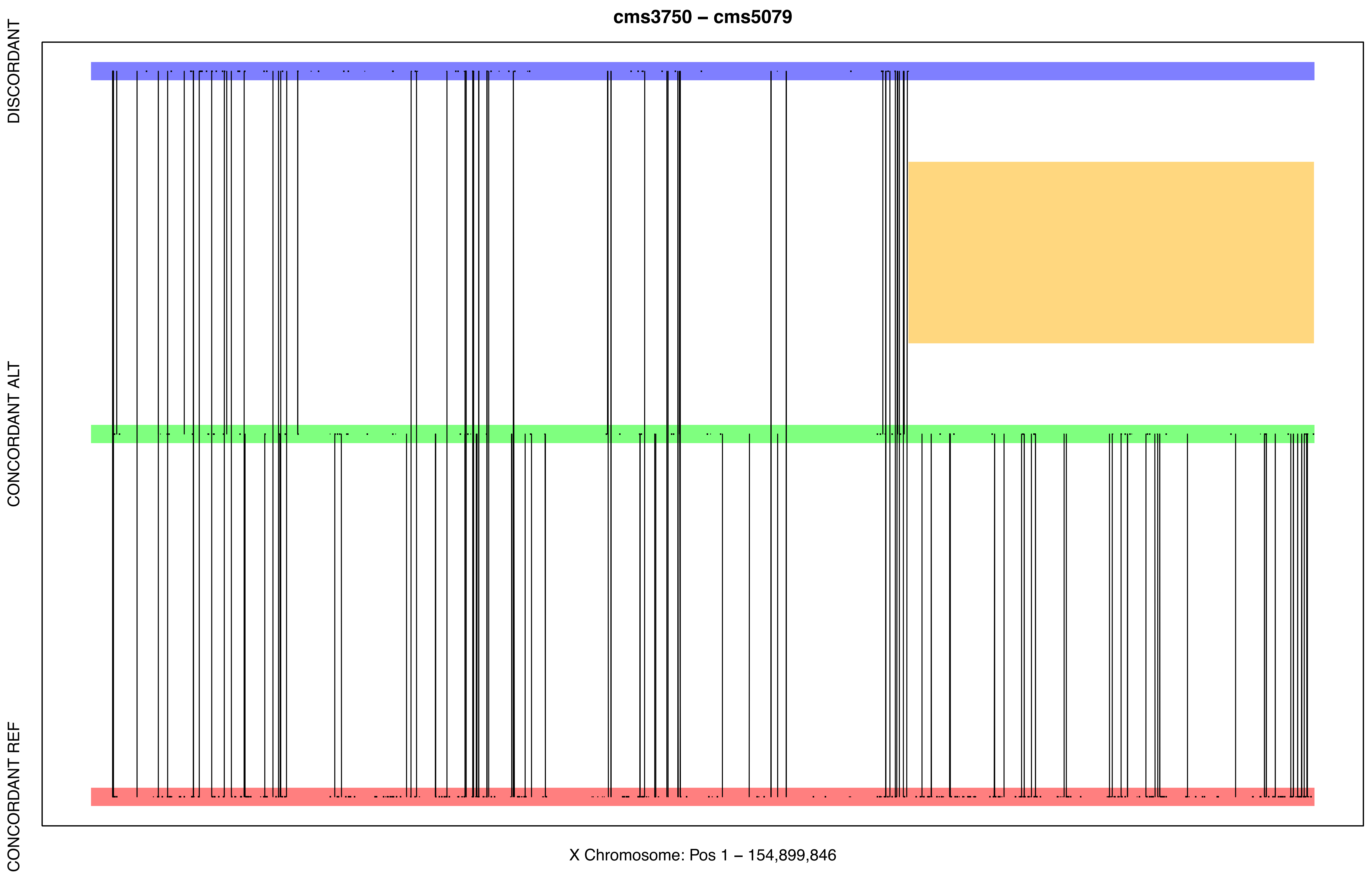

cms3750 – cms5079
DISCORDANT
CONCORDANT ALT
CONCORDANT REF
X Chromosome: Pos 1 – 154,899,846

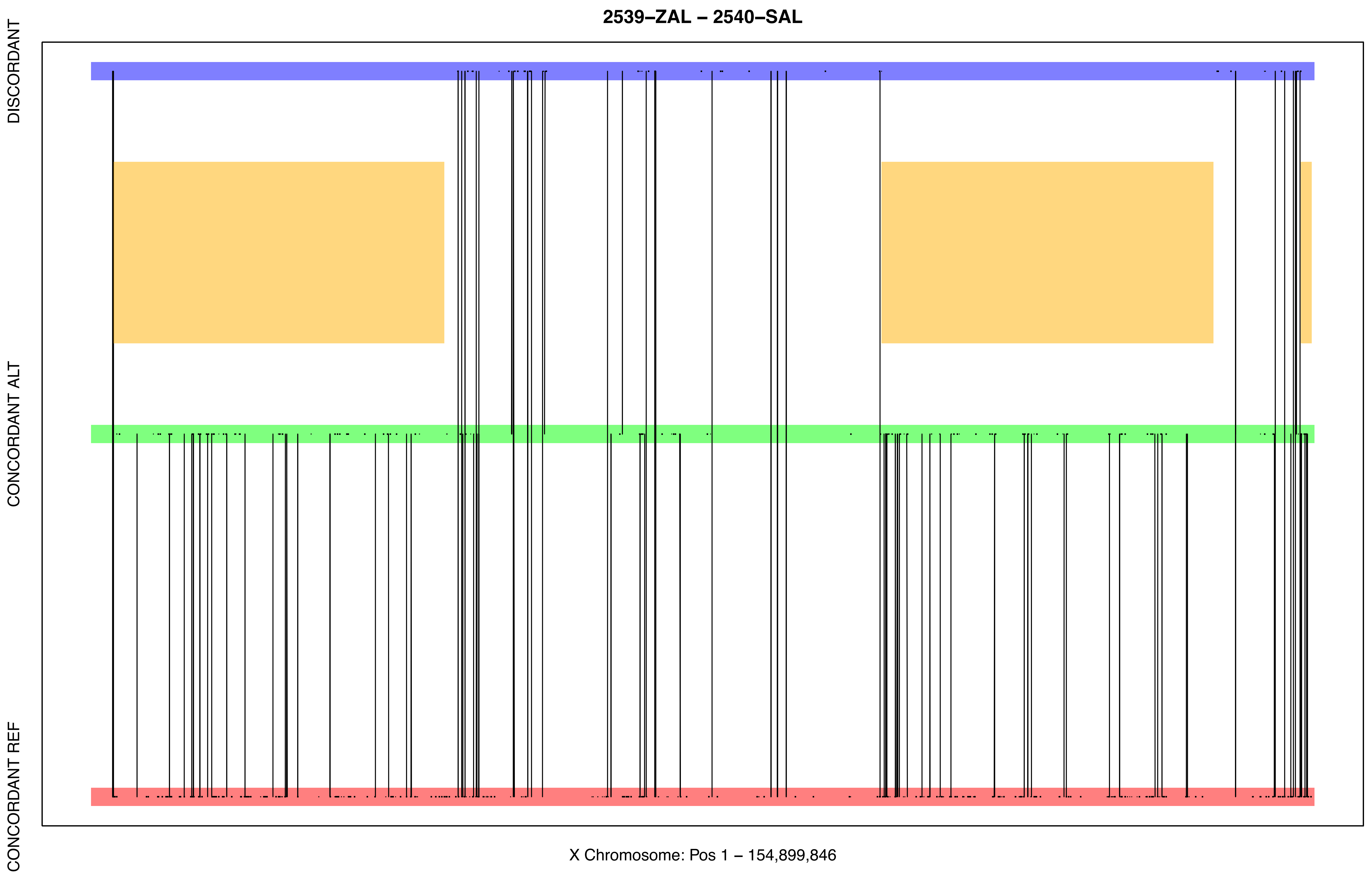

2539–ZAL – 2540–SAL
DISCORDANT
CONCORDANT ALT
CONCORDANT REF
X Chromosome: Pos 1 – 154,899,846

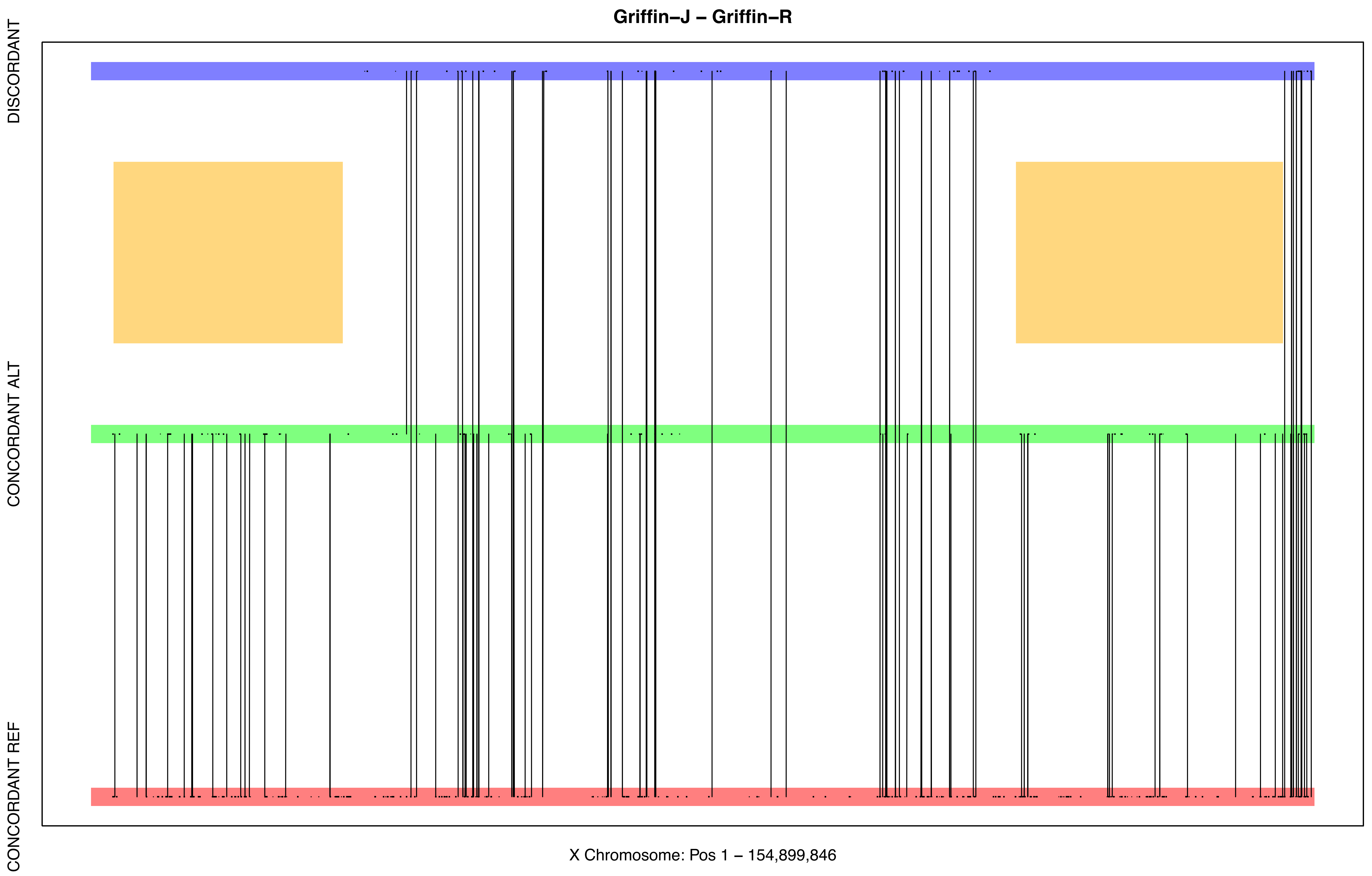
Griffin–J – Griffin–R
DISCORDANT
CONCORDANT ALT
CONCORDANT REF
X Chromosome: Pos 1 – 154,899,846

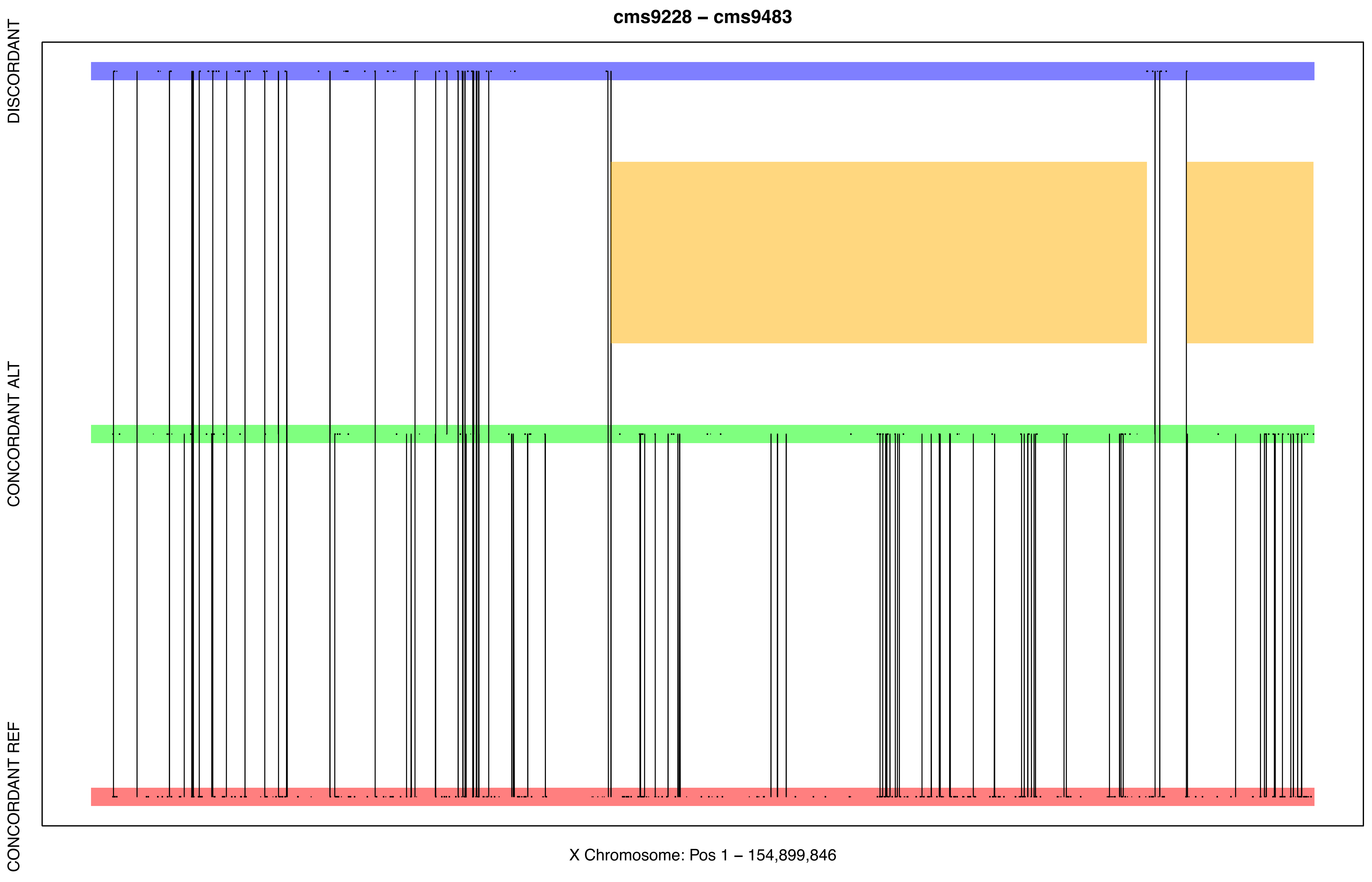

cms9228 – cms9483
DISCORDANT
CONCORDANT ALT
CONCORDANT REF
X Chromosome: Pos 1 – 154,899,846

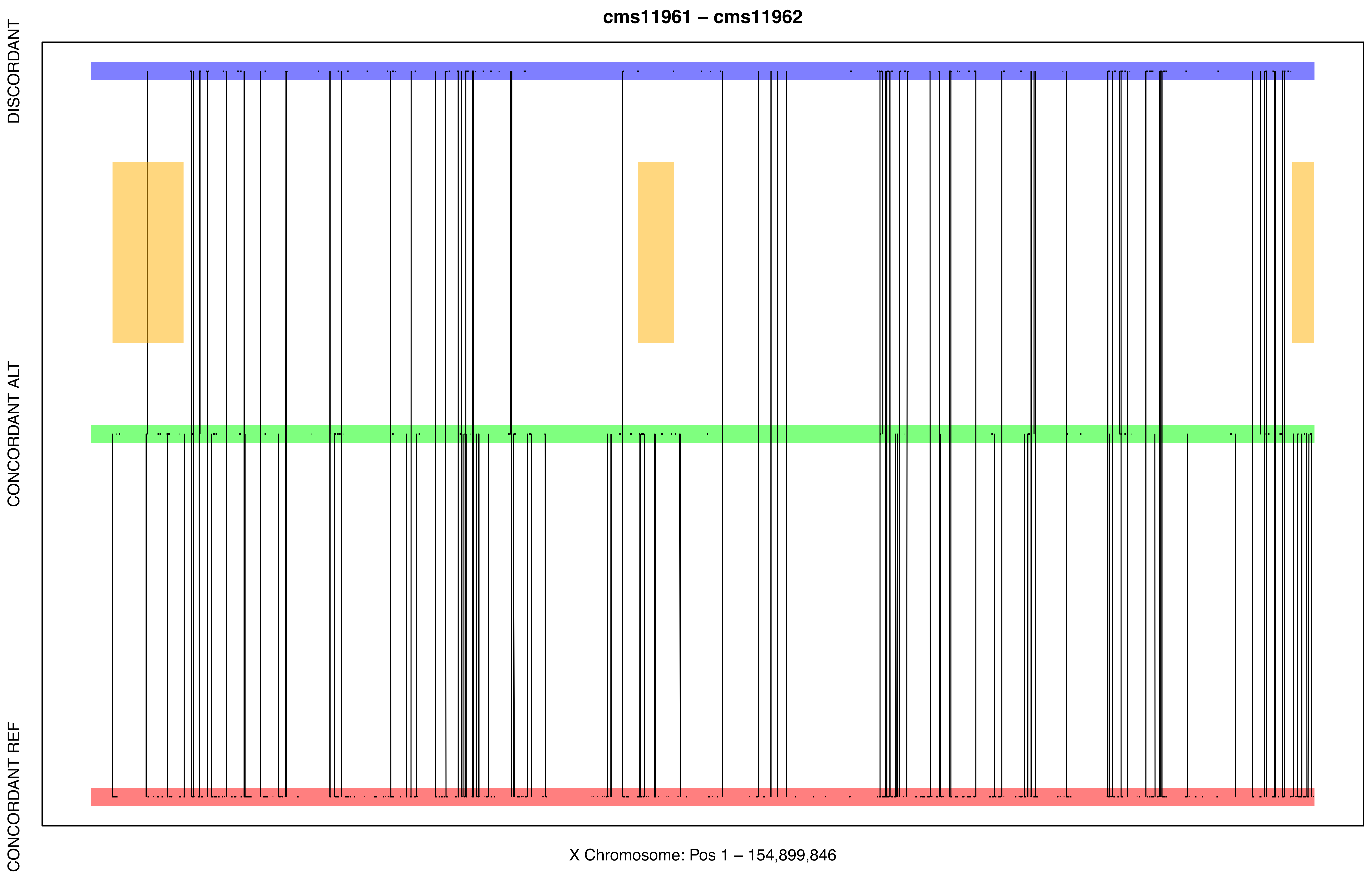

cms11961 – cms11962
DISCORDANT
CONCORDANT ALT
CONCORDANT REF
X Chromosome: Pos 1 – 154,899,846

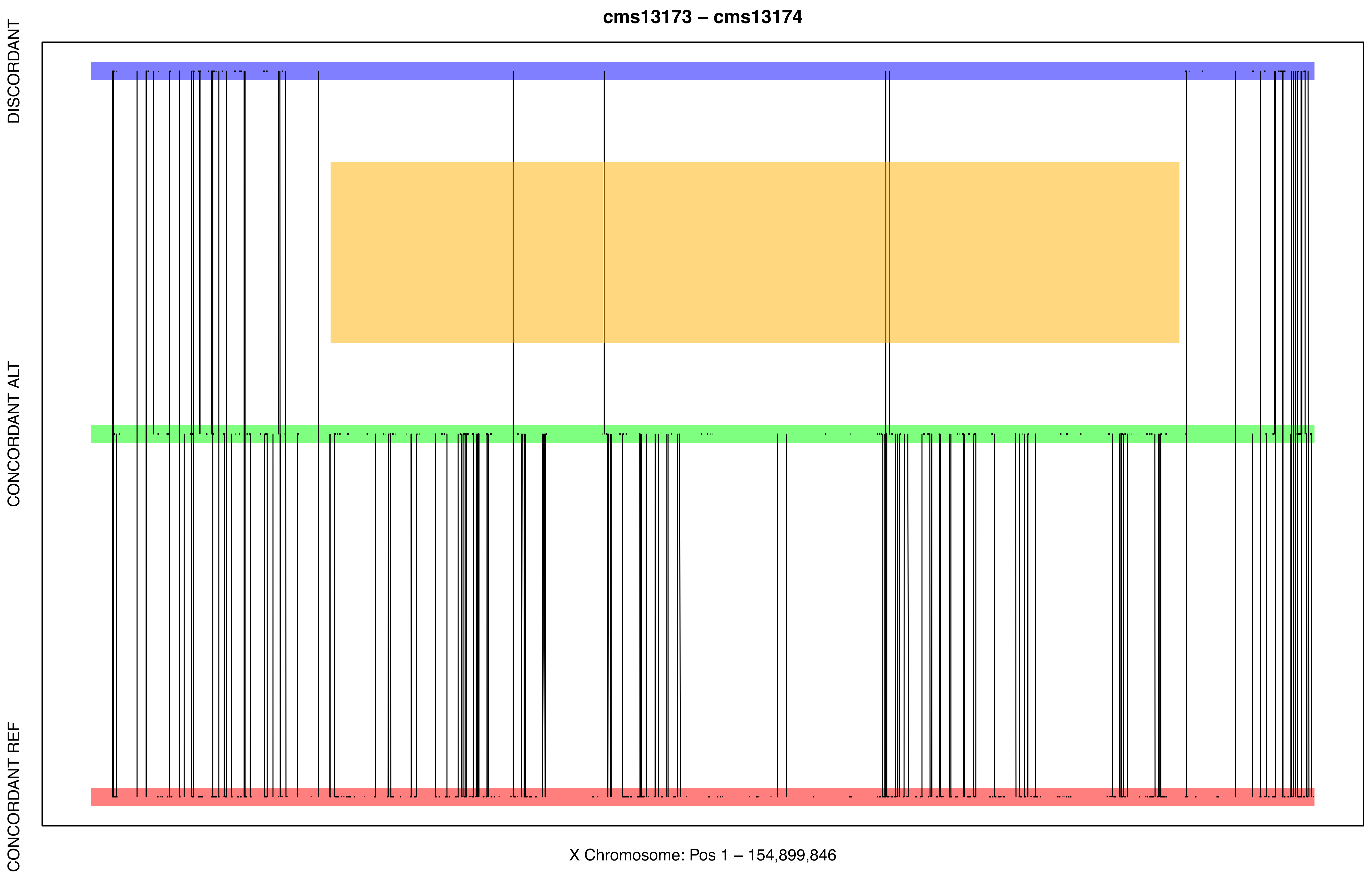
cms13173 – cms13174
DISCORDANT
CONCORDANT ALT
CONCORDANT REF
X Chromosome: Pos 1 – 154,899,846

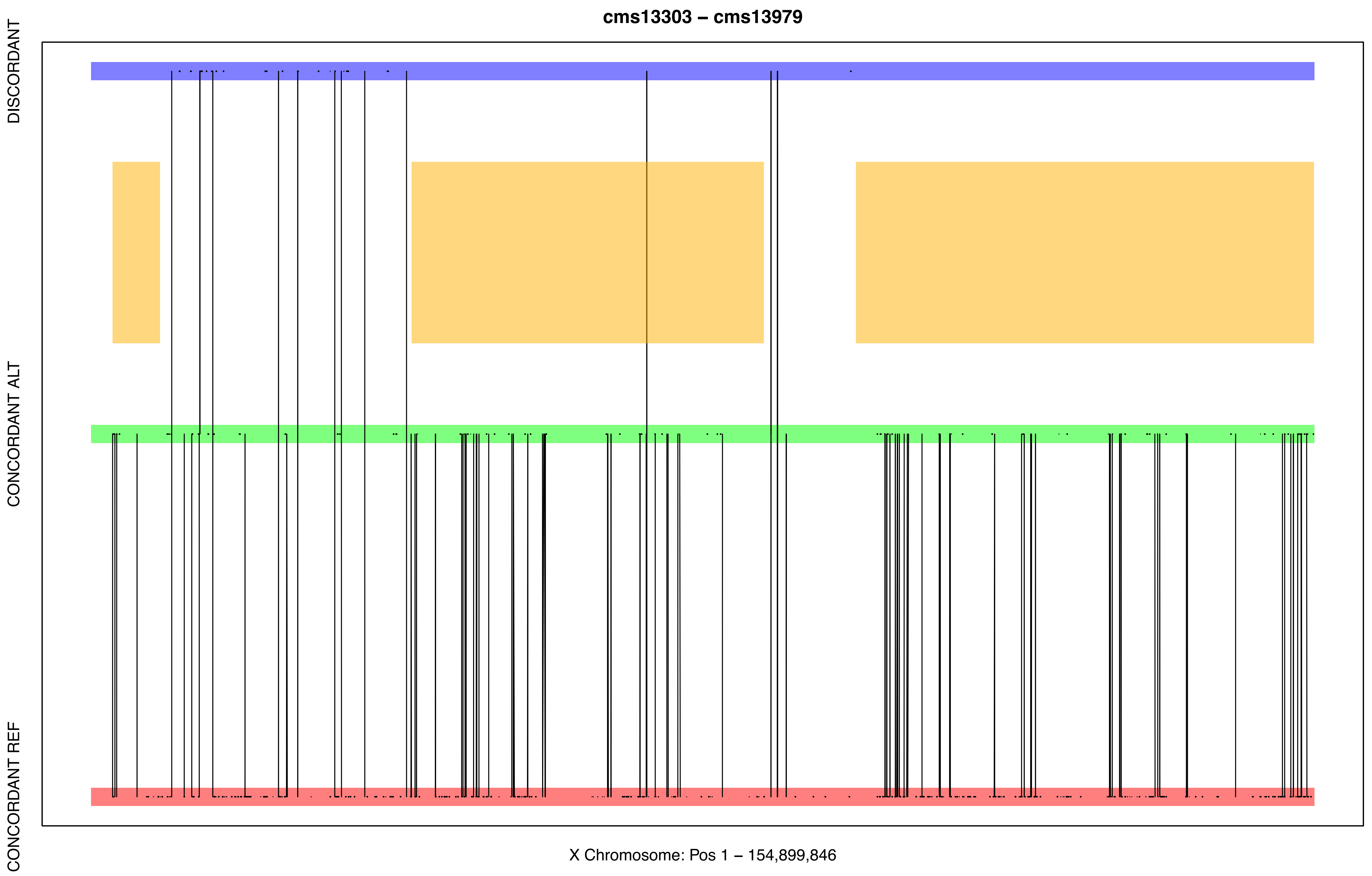
cms13303 – cms13979
DISCORDANT
CONCORDANT ALT
CONCORDANT REF
X Chromosome: Pos 1 – 154,899,846

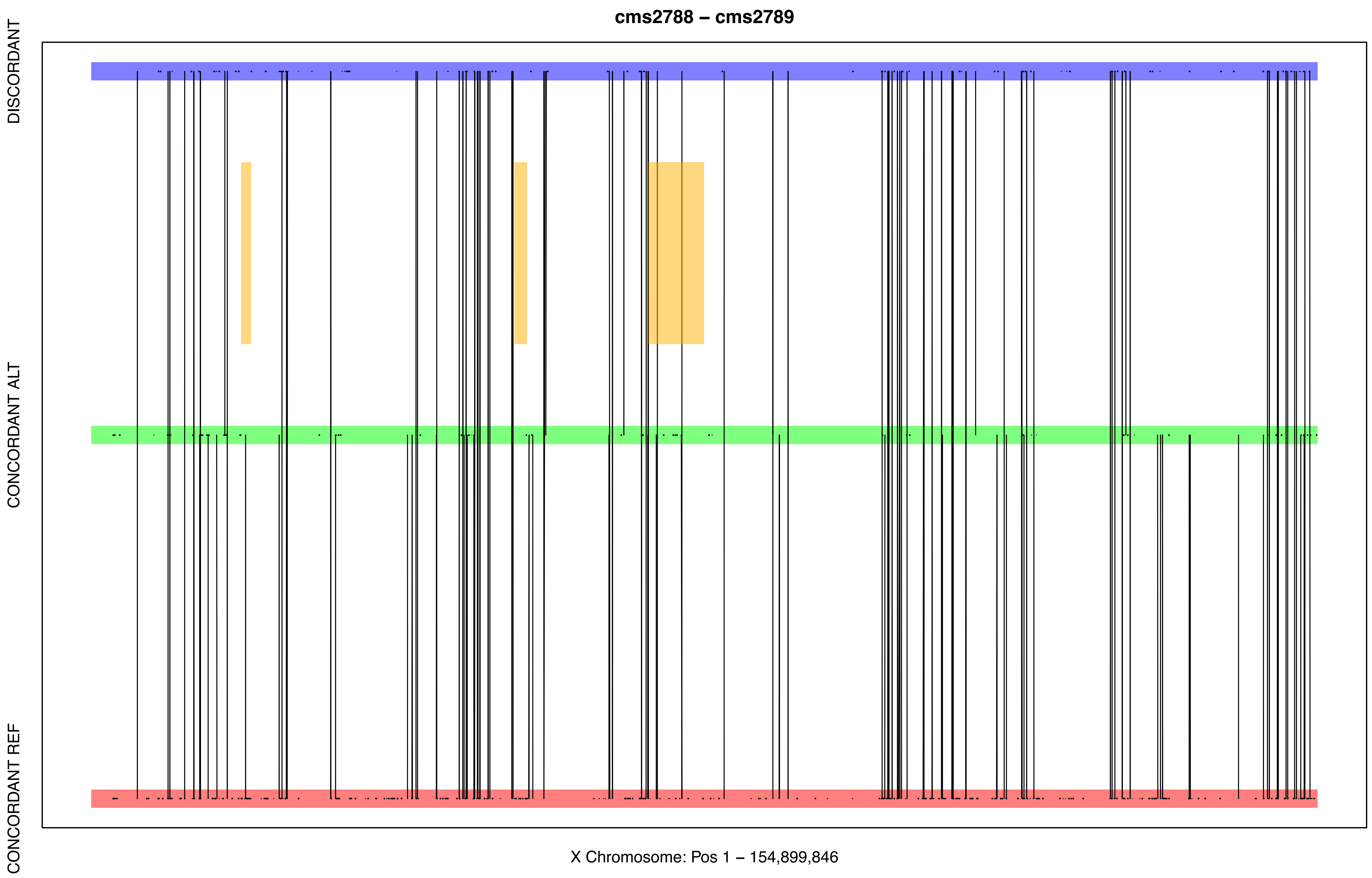

cms2788 – cms2789
DISCORDANT
CONCORDANT ALT
CONCORDANT REF
X Chromosome: Pos 1 – 154,899,846

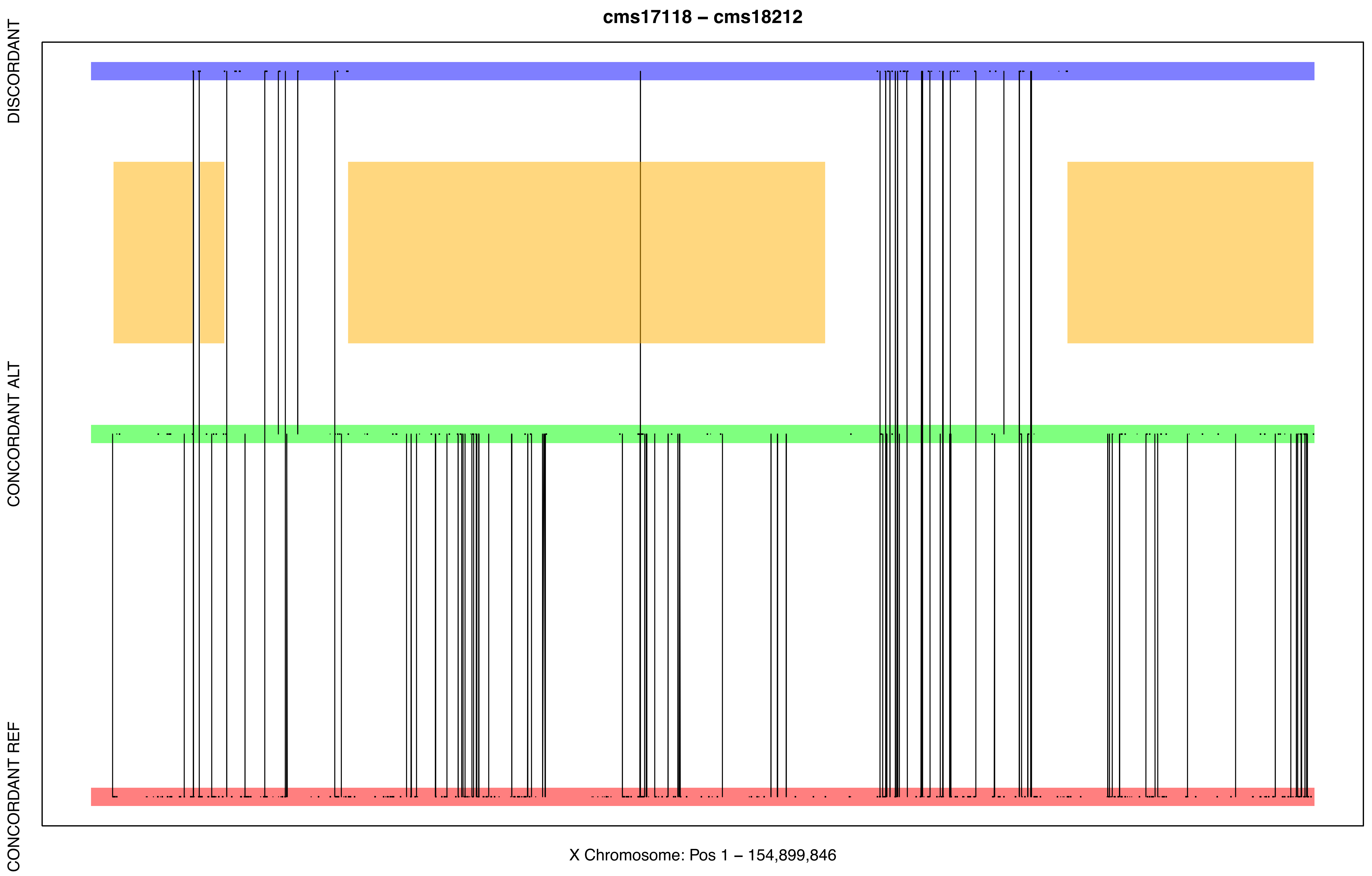
cms17118 – cms18212
DISCORDANT
CONCORDANT ALT
CONCORDANT REF
X Chromosome: Pos 1 – 154,899,846

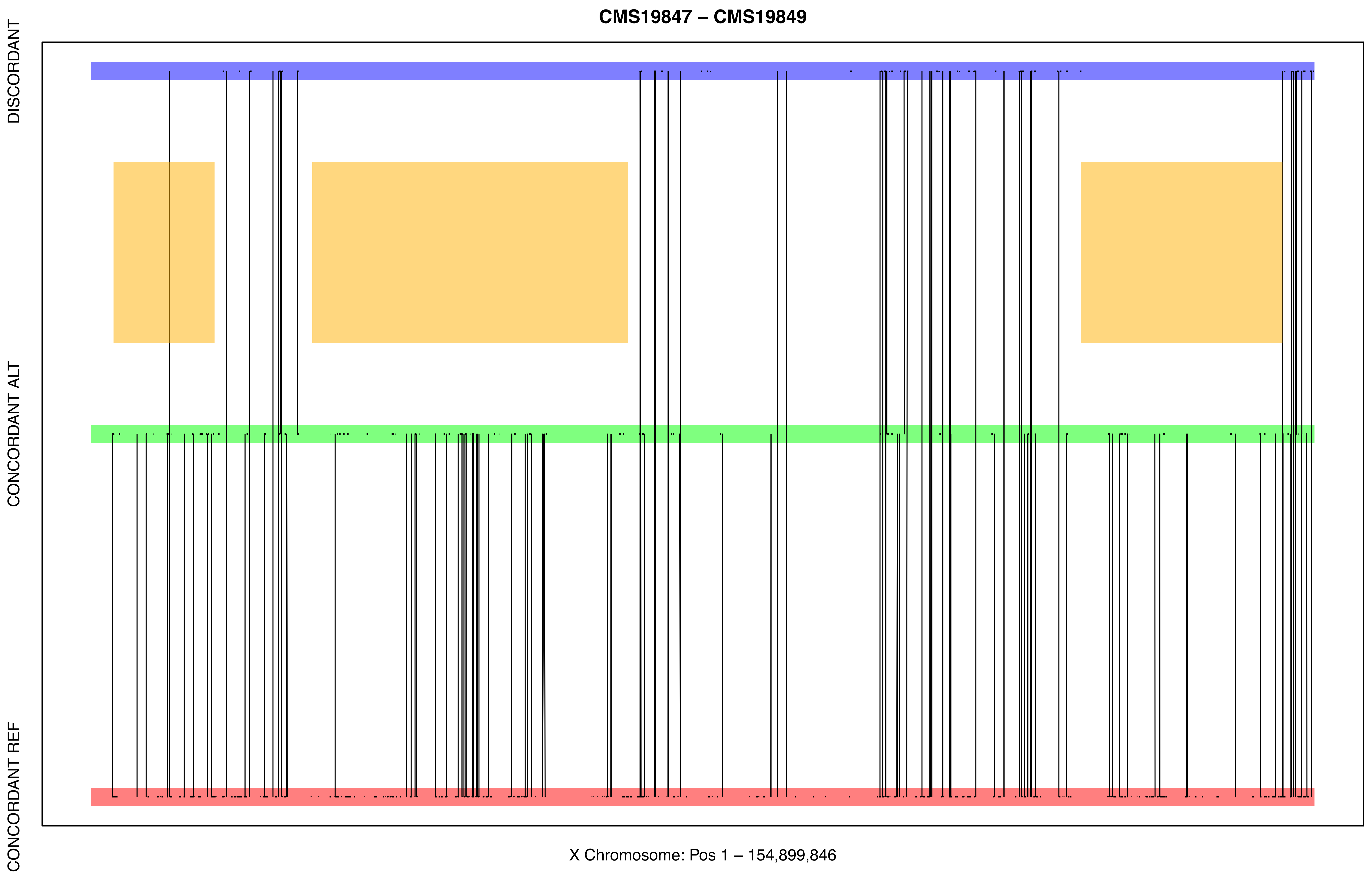

CMS19847 – CMS19849
DISCORDANT
CONCORDANT ALT
CONCORDANT REF
X Chromosome: Pos 1 – 154,899,846

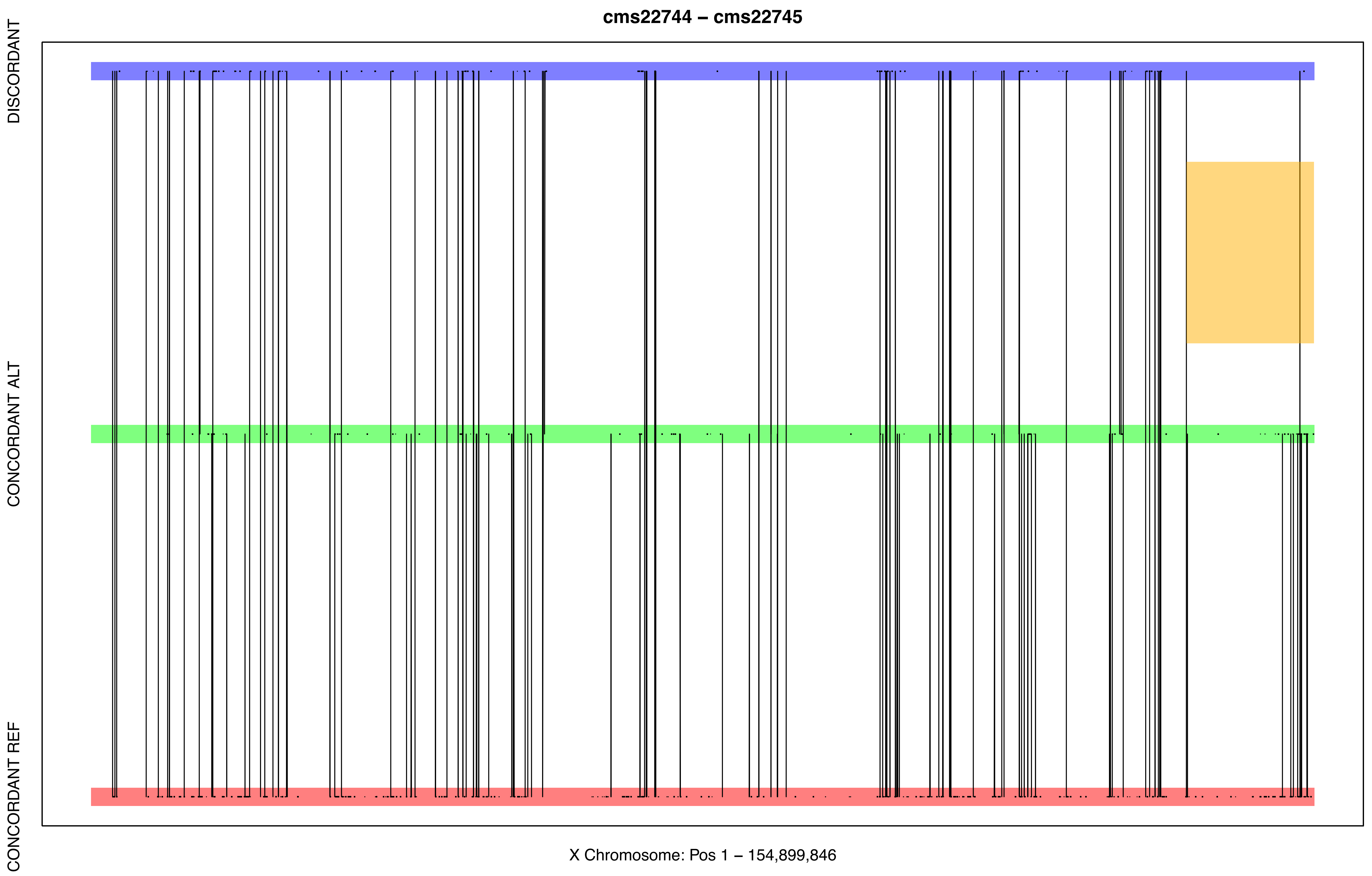

cms22744 – cms22745
DISCORDANT
CONCORDANT ALT
CONCORDANT REF
X Chromosome: Pos 1 – 154,899,846

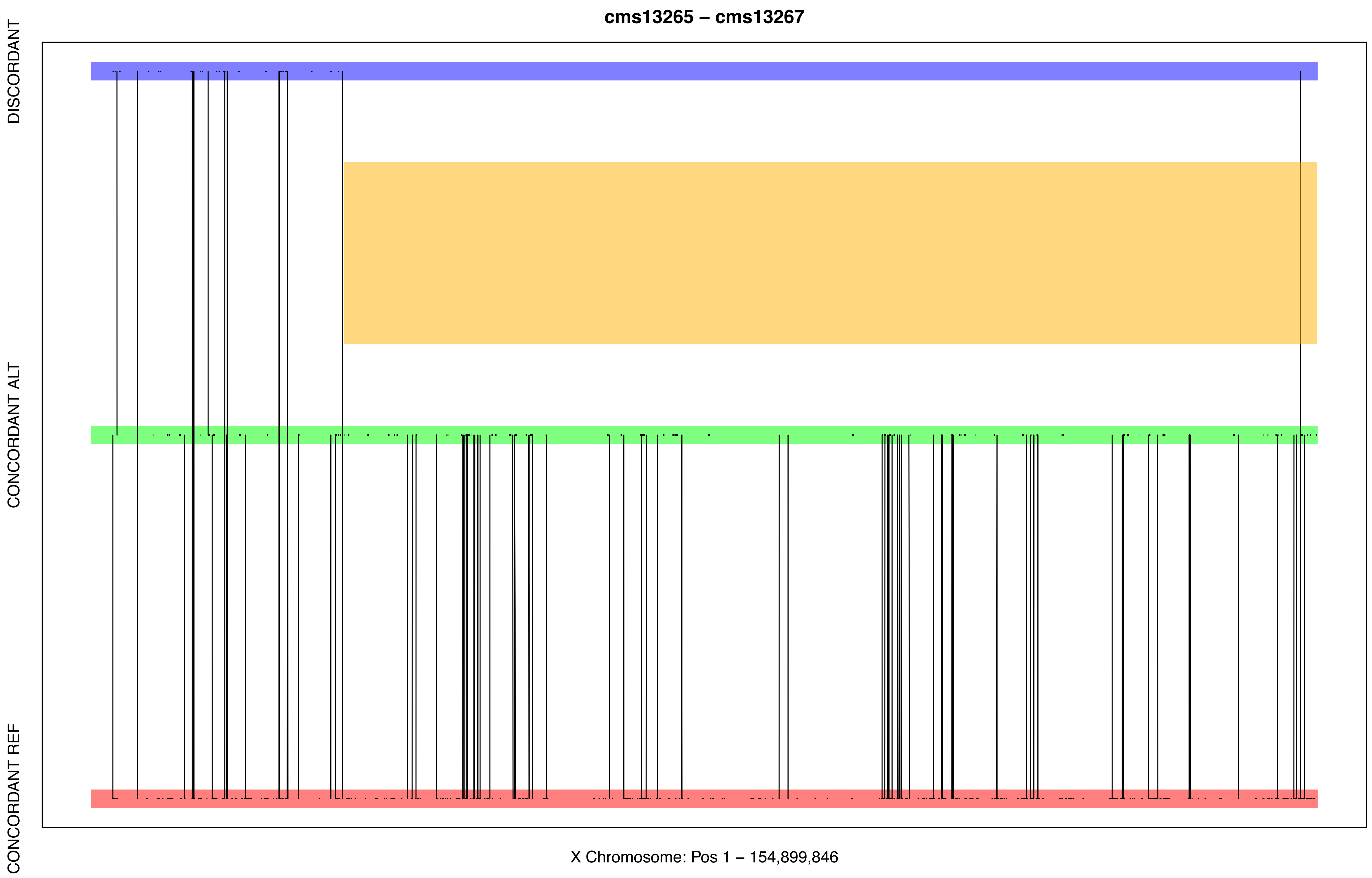
cms13265 – cms13267
DISCORDANT
CONCORDANT ALT
CONCORDANT REF
X Chromosome: Pos 1 – 154,899,846

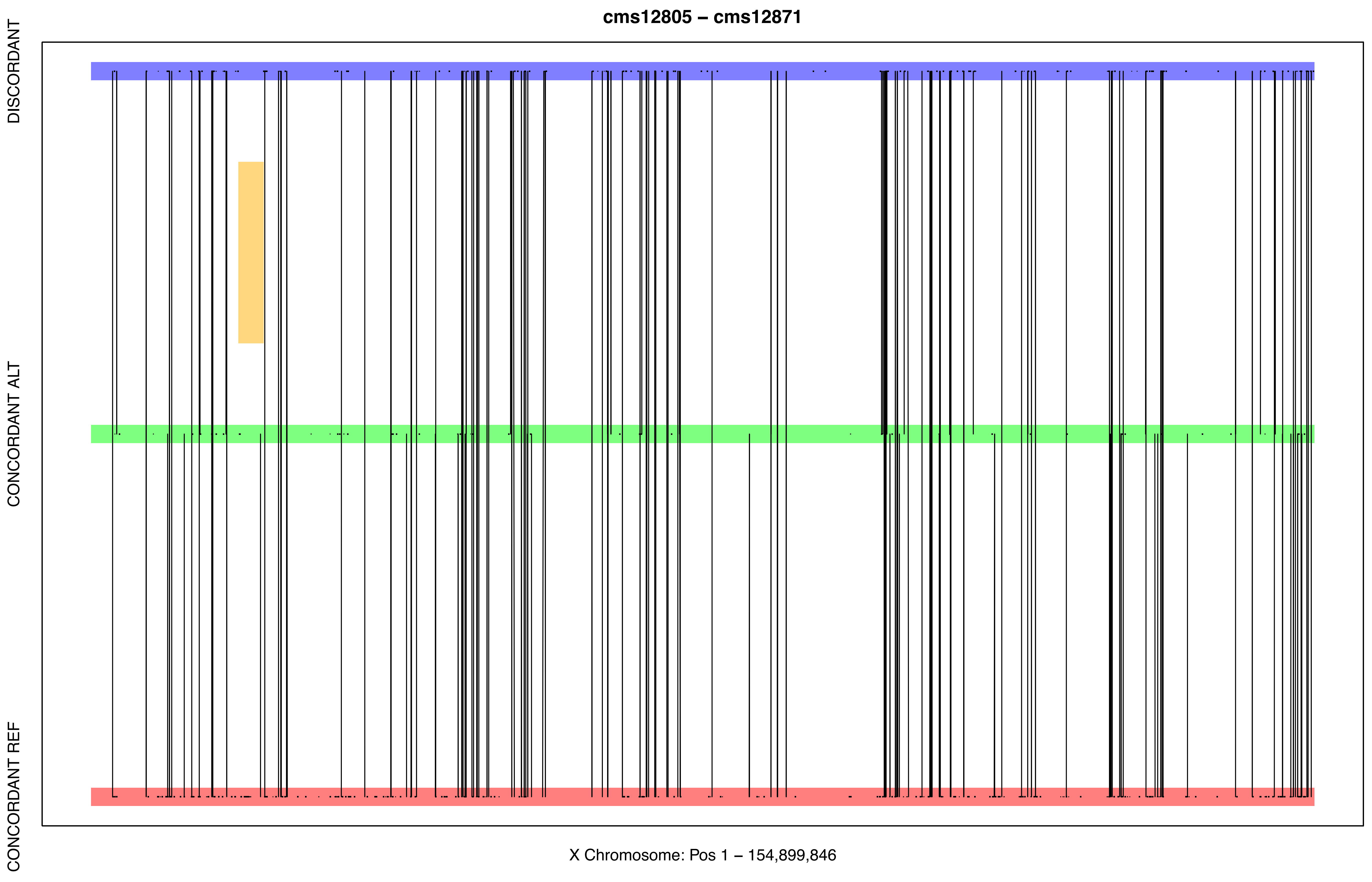
cms12805 – cms12871
DISCORDANT
CONCORDANT ALT
CONCORDANT REF
X Chromosome: Pos 1 – 154,899,846

13439 – cms1861

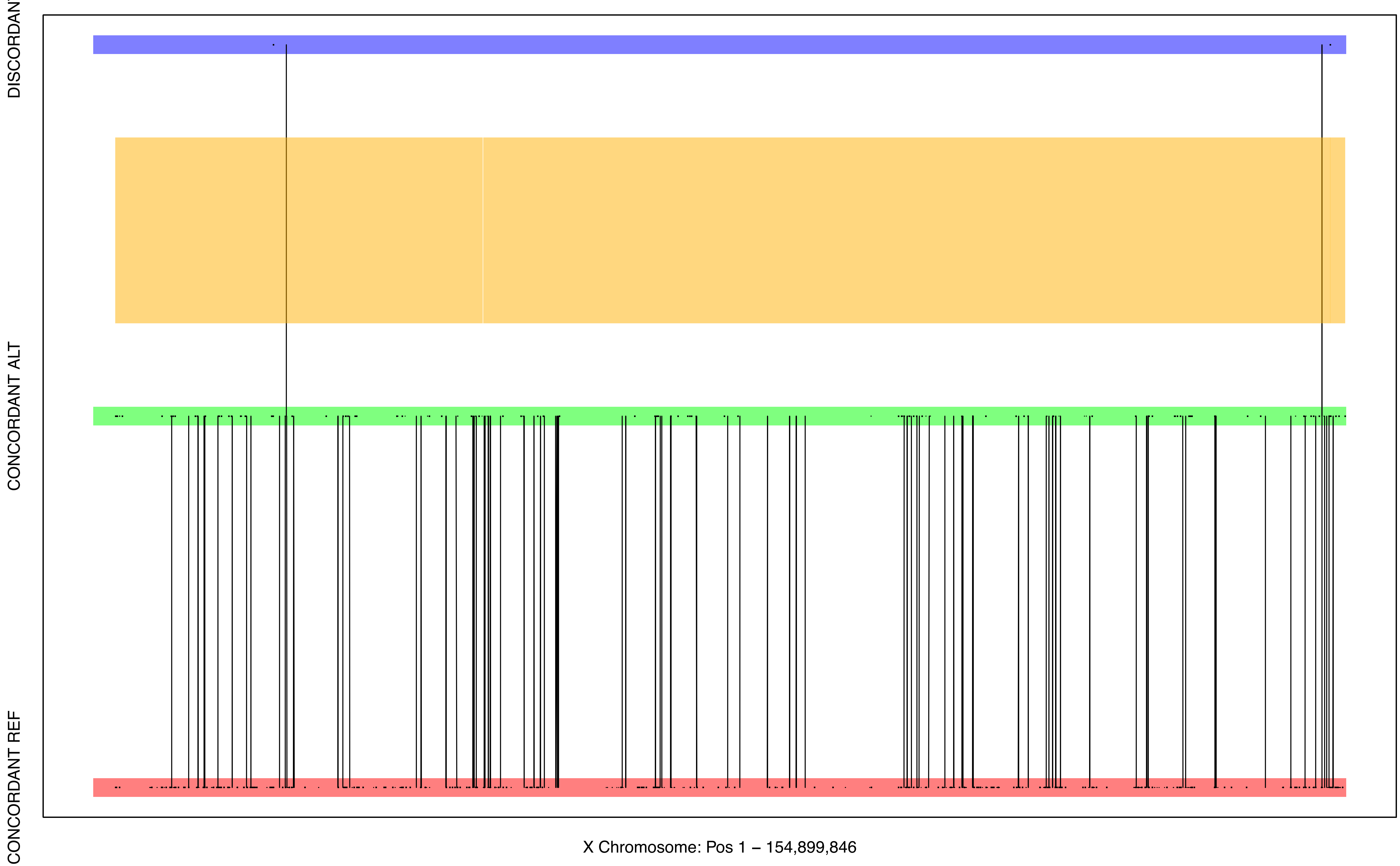

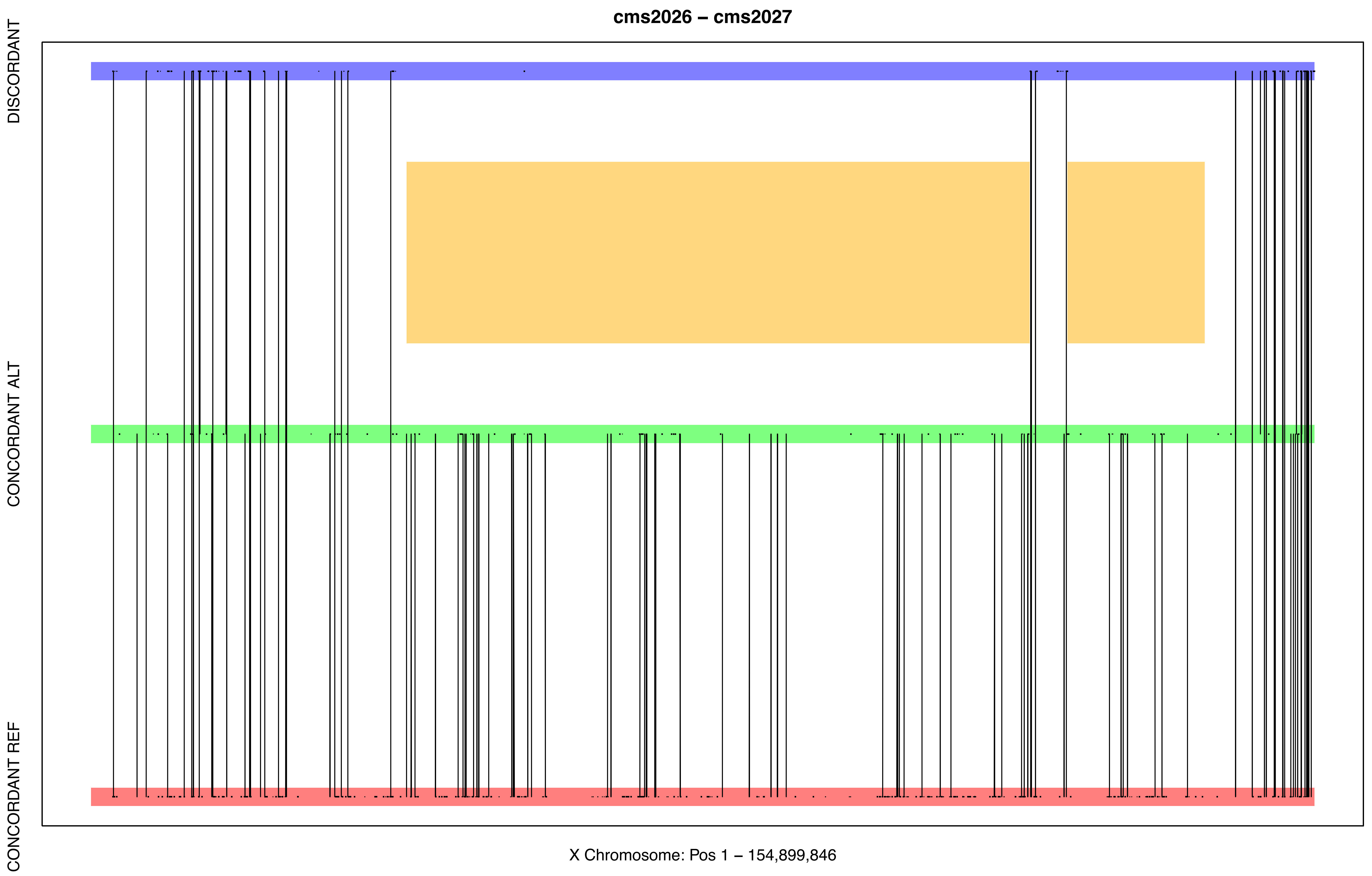

cms2026 – cms2027
DISCORDANT
CONCORDANT ALT
CONCORDANT REF
X Chromosome: Pos 1 – 154,899,846

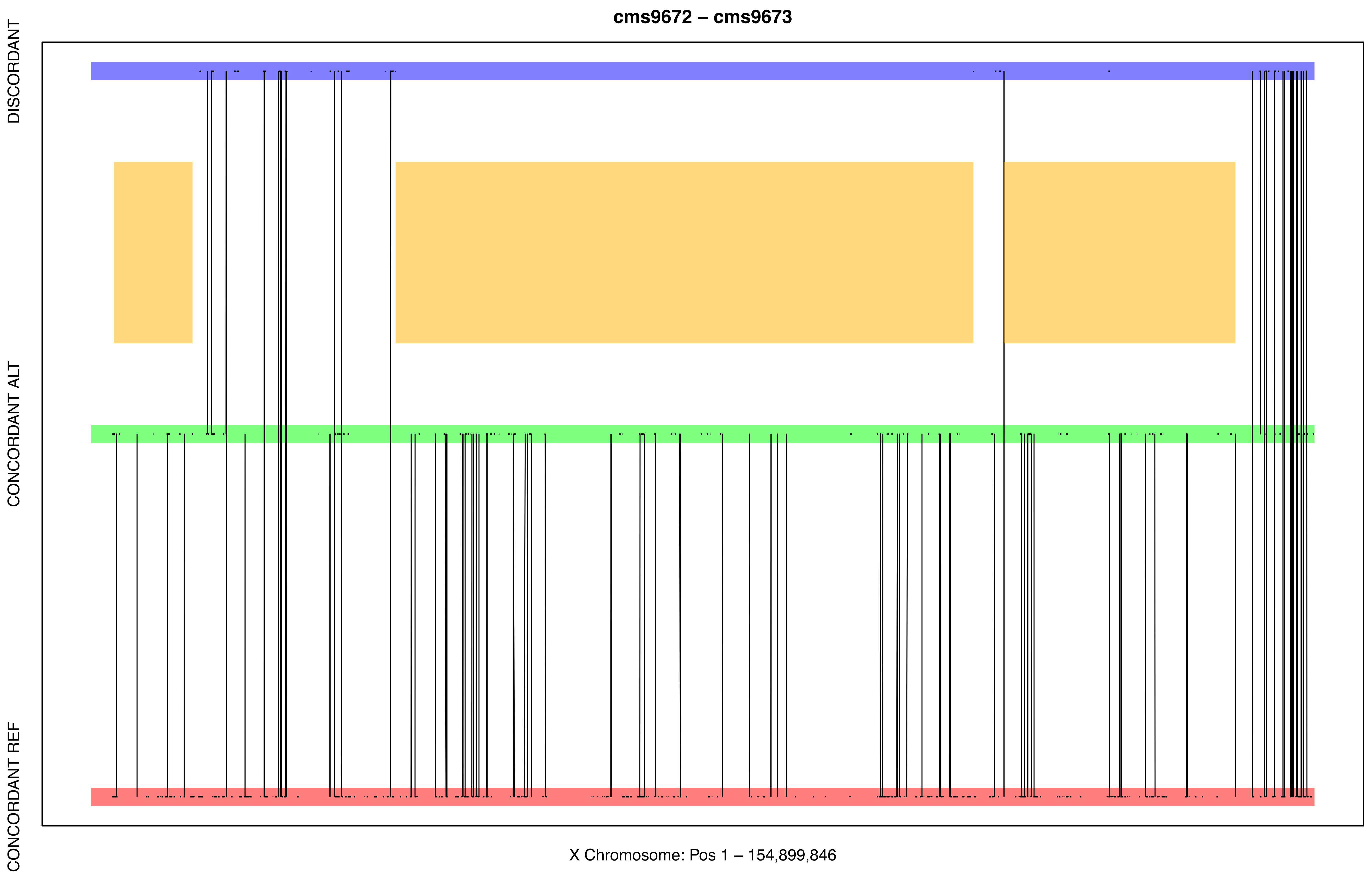

cms9672 – cms9673
DISCORDANT
CONCORDANT ALT
CONCORDANT REF
X Chromosome: Pos 1 – 154,899,846

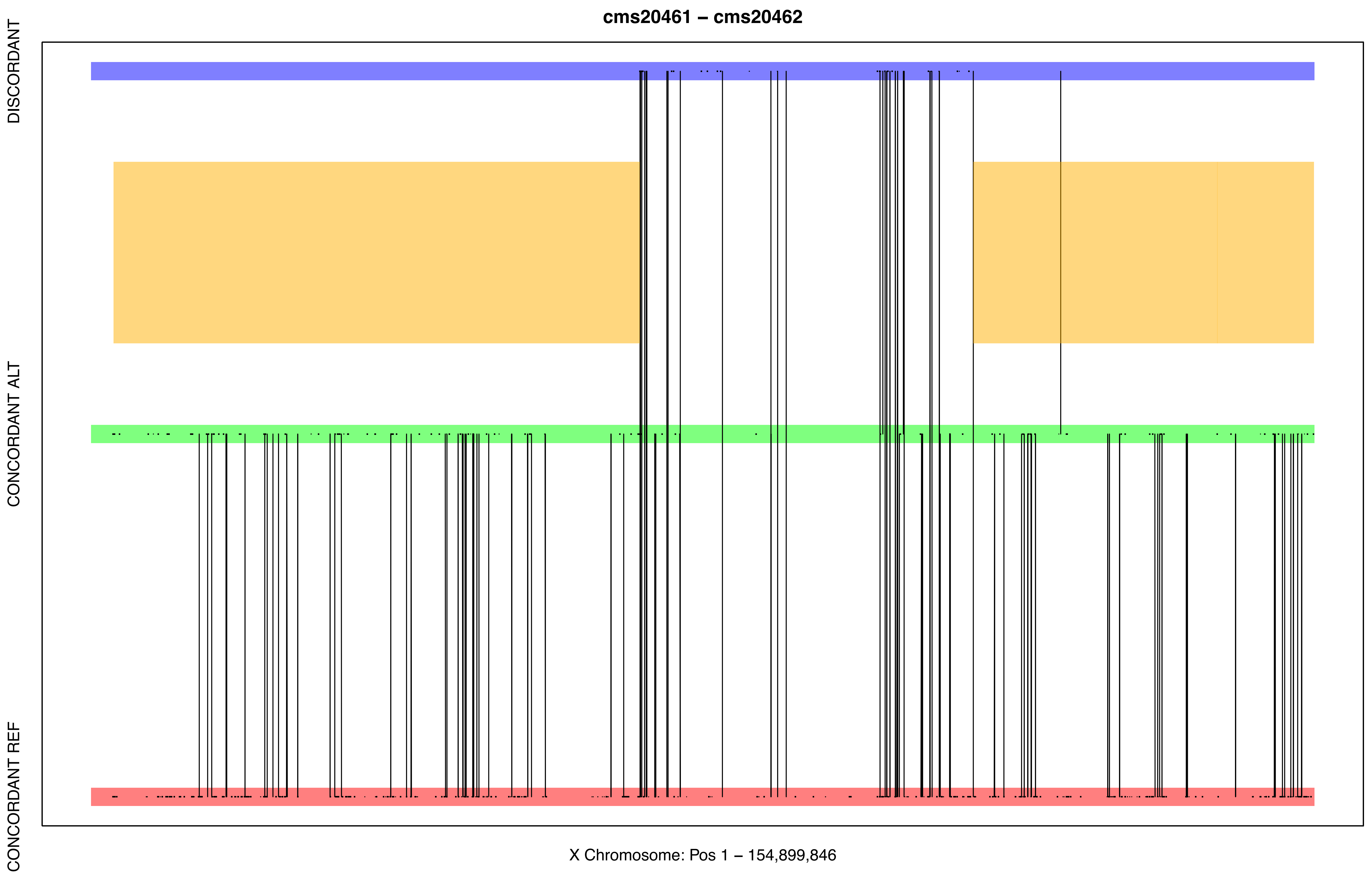

cms20461 – cms20462
DISCORDANT
CONCORDANT ALT
CONCORDANT REF
X Chromosome: Pos 1 – 154,899,846

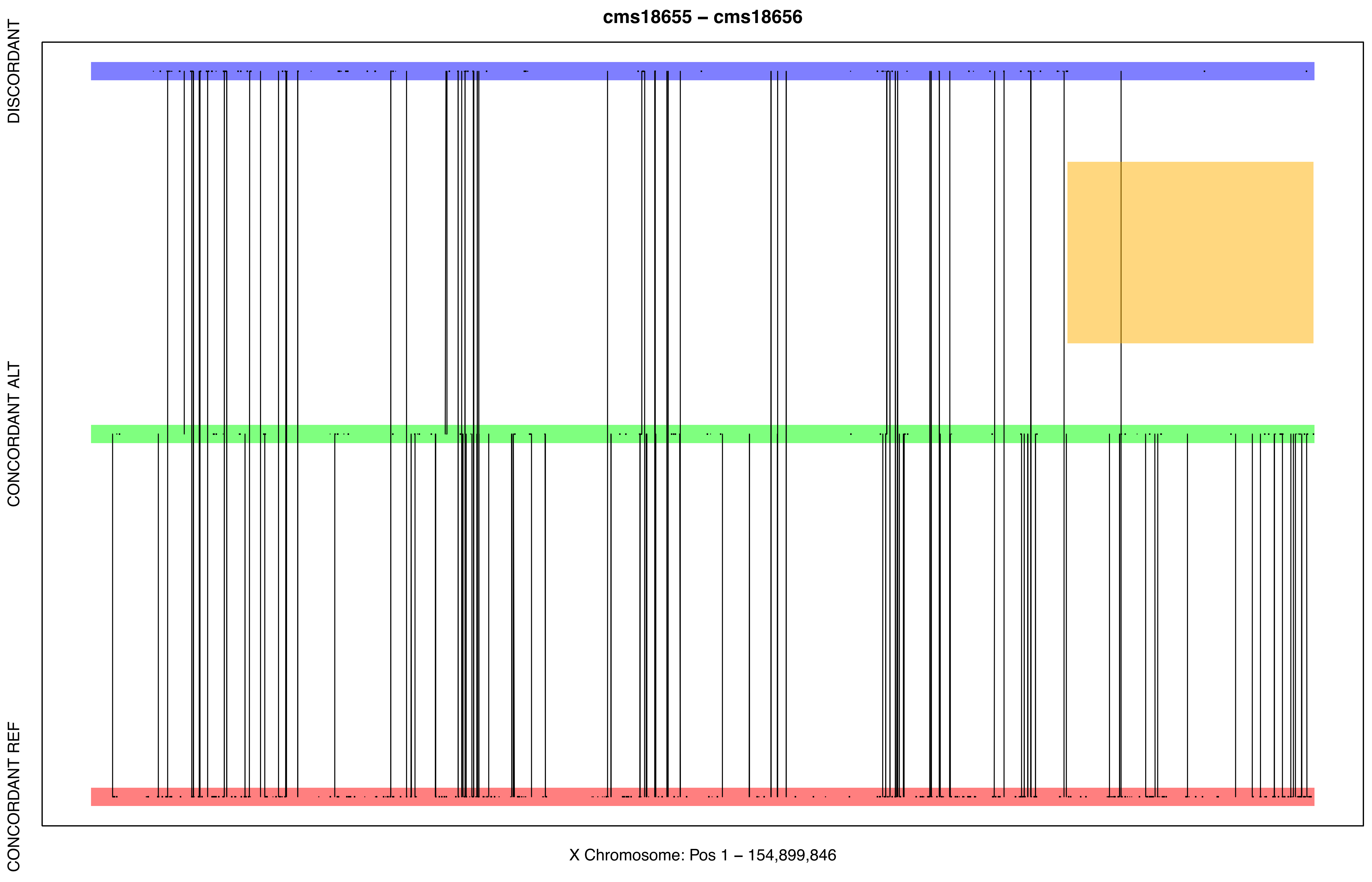

cms18655 – cms18656
DISCORDANT
CONCORDANT ALT
CONCORDANT REF
X Chromosome: Pos 1 – 154,899,846

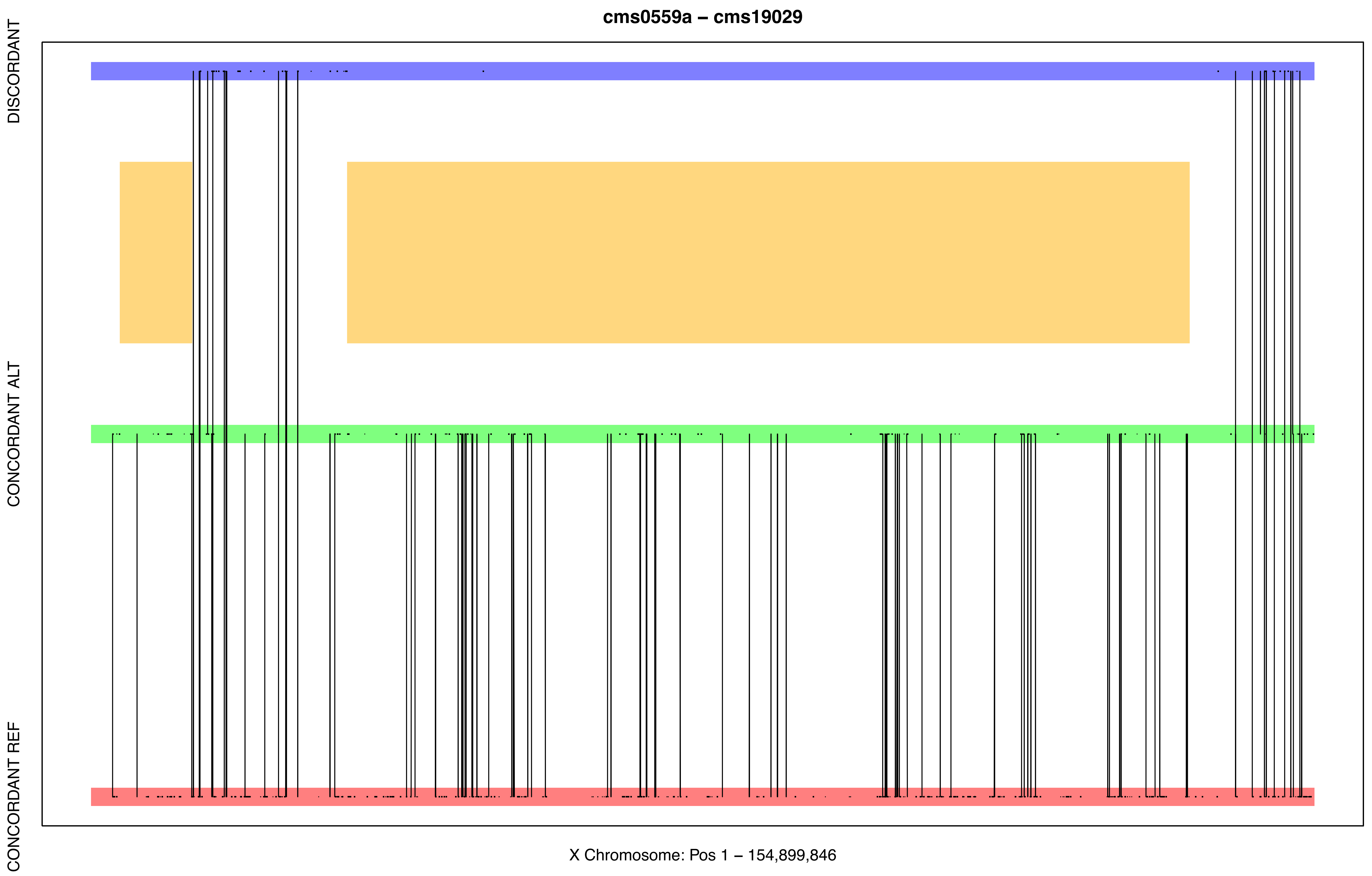
cms0559a – cms19029
DISCORDANT
CONCORDANT ALT
CONCORDANT REF
X Chromosome: Pos 1 – 154,899,846

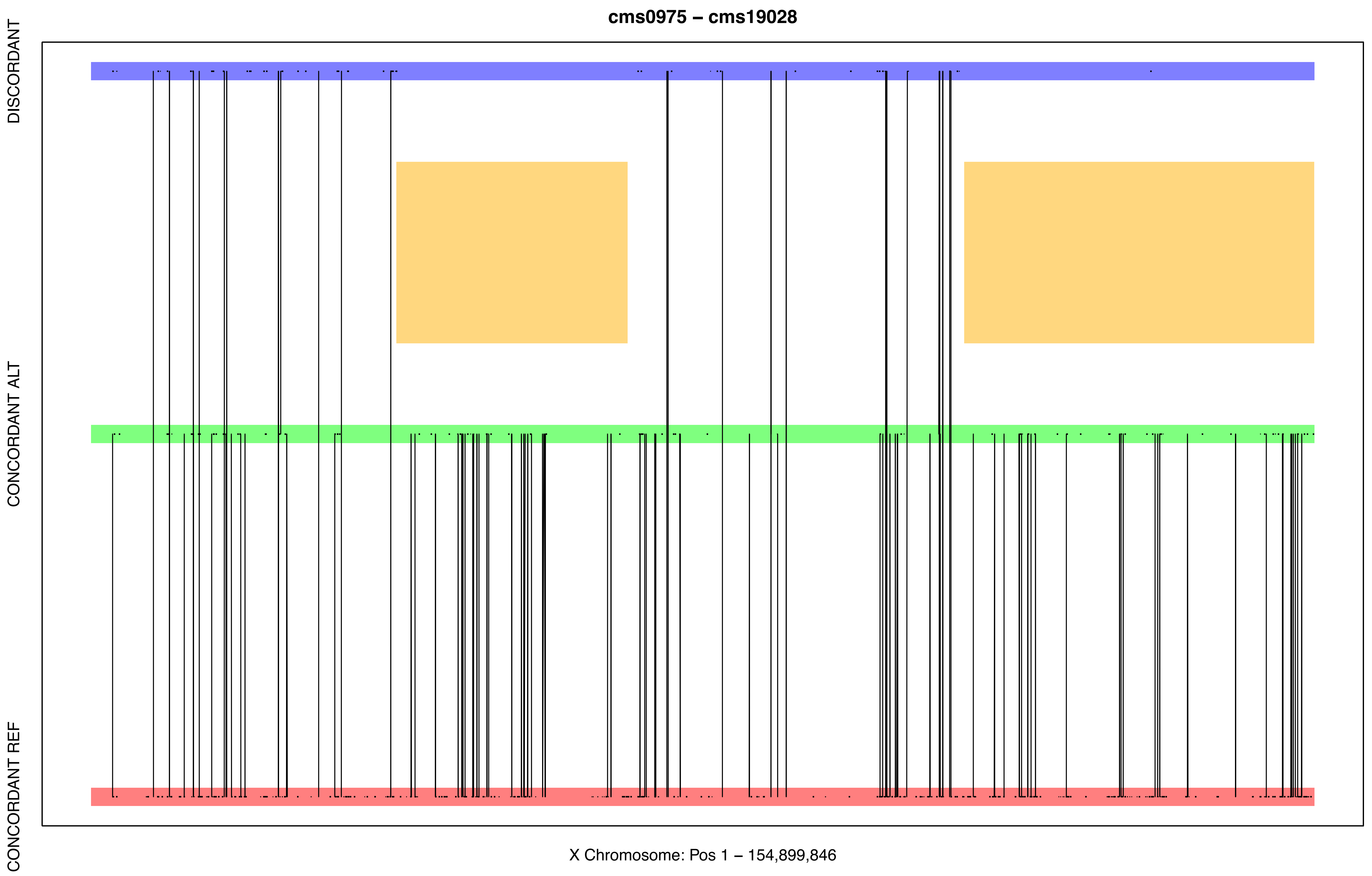
cms0975 – cms19028
DISCORDANT
CONCORDANT ALT
CONCORDANT REF
X Chromosome: Pos 1 – 154,899,846

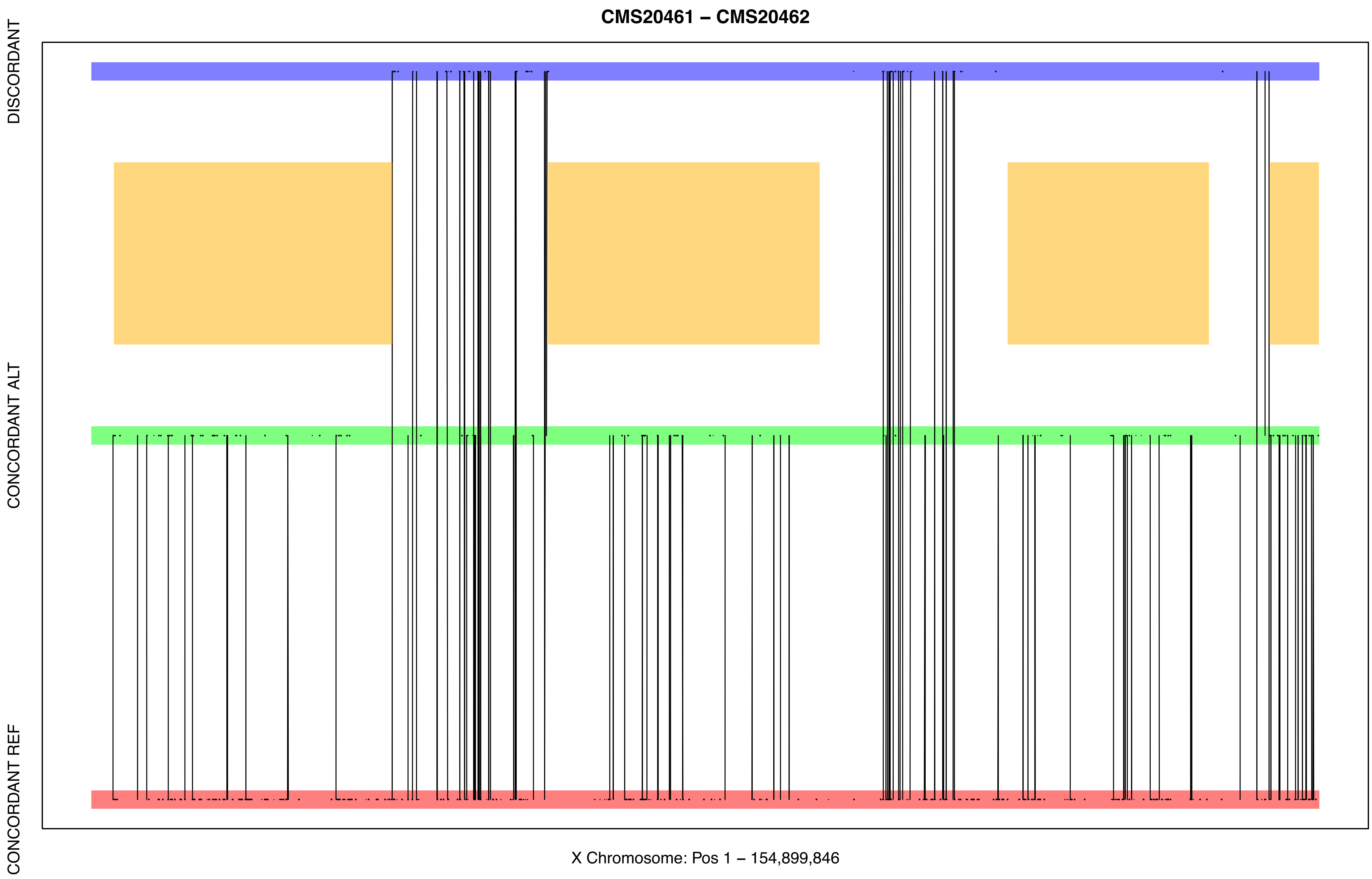
CMS20461 – CMS20462
DISCORDANT
CONCORDANT ALT
CONCORDANT REF
X Chromosome: Pos 1 – 154,899,846
