# Supplementary material for: Affected Kindred Analysis of Human X Chromosome Exomes to Identify Novel X-Linked Intellectual Disability Genes
Source: PLoS One. 2015 Feb 13;10(2):e0116454. doi: 10.1371/journal.pone.0116454 (PMC4332666; doi:10.1371/journal.pone.0116454)
Supplement: S3 Fig — Population stratification was determined by comparing samples to a cohort mean, and by comparing sample allele frequencies to population-specific allele frequencies derived from EVS data. For the cohort mean analysis, an average allele frequency for the cohort was obtained for each variant. A residual sum of squares was subsequently calculated for each sample compared to the cohort mean. The number of standard deviations from the residual is plotted on the Y-axis of S3a Fig.. Comparison of sample allele frequencies to EVS was performed for both EVS European American (EA) and EVS African American (AA) population frequencies. For any given sample, an EA and an AA metric was obtained by averaging the population specific allele frequencies for all sample variants present in the EVS dataset. This EA metric for each sample is plotted on the X-axis of S3 Fig. The AA metric for each sample is plotted on the Y-axis of S3b Fig. The majority of samples cluster together with genotypes of primarily European ancestry. However, five samples show slight deviations from the main cluster. When SNP loads were compared to EVS data from the African American population, the sample cluster deviation was reproduced, indicating that these five samples have a small, but detectable contribution of African ancestry. (PDF) [file pone.0116454.s003.pdf]

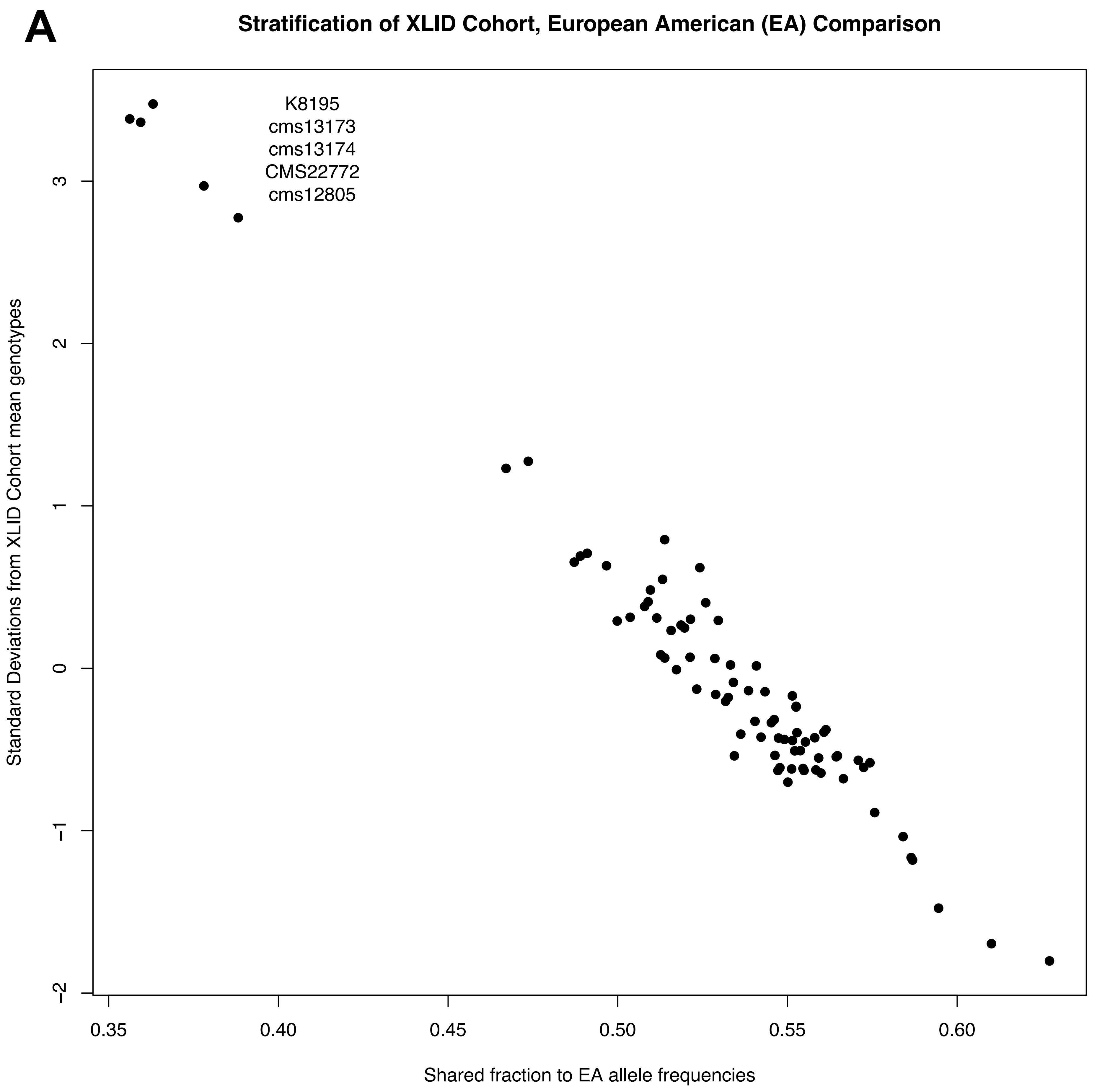
A
Stratification of XLID Cohort, European American (EA) Comparison
K8195
cms13173
cms13174
CMS22772
cms12805
Standard Deviations from XLID Cohort mean genotypes
−2
−1
0
1
2
3
0.35
0.40
0.45
0.50
0.55
0.60
Shared fraction to EA allele frequencies

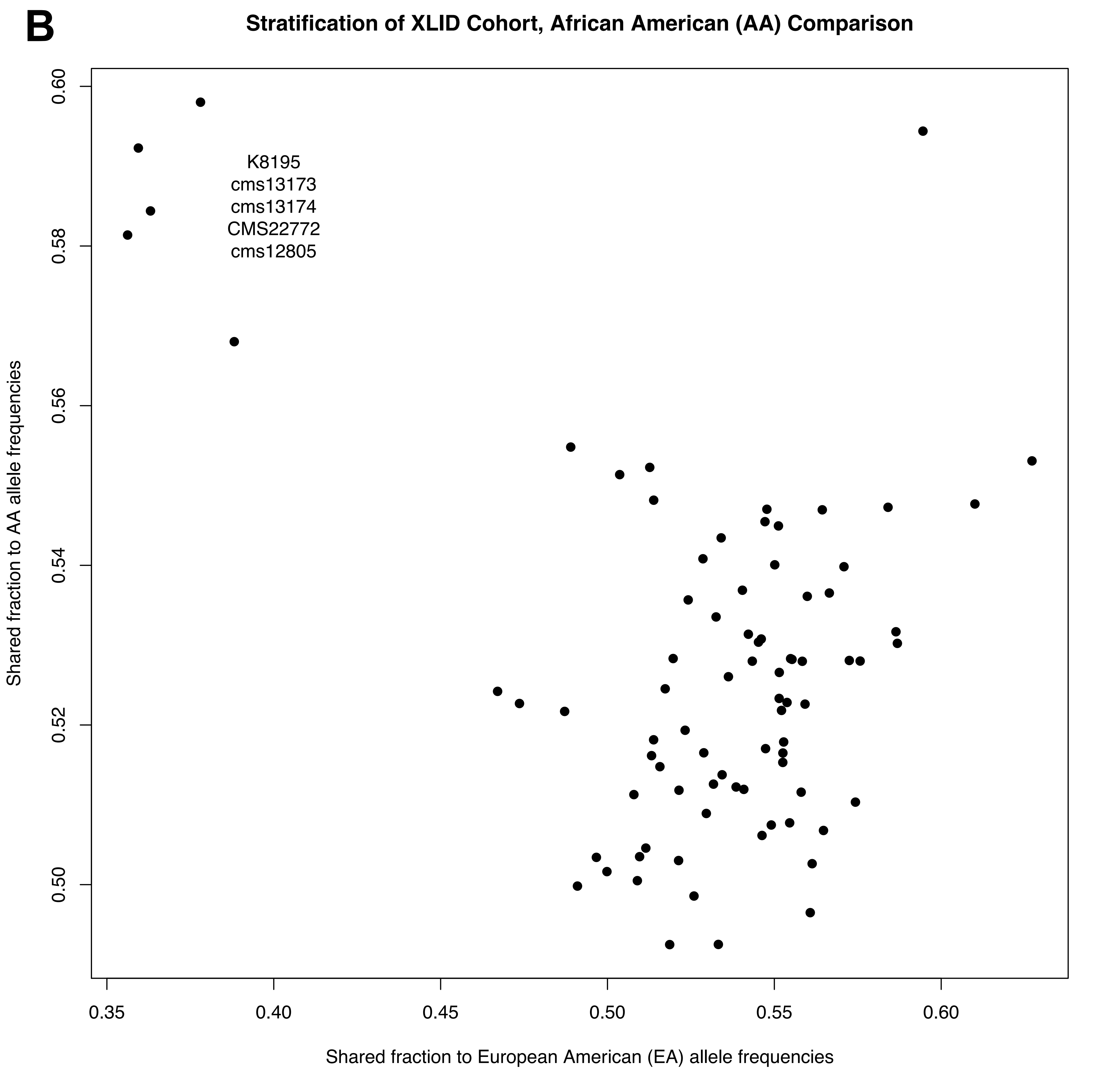

B
Stratification of XLID Cohort, African American (AA) Comparison
K8195
cms13173
cms13174
CMS22772
cms12805
0.60
0.58
0.56
0.54
0.52
0.50
0.35
0.40
0.45
0.50
0.55
0.60
Shared fraction to AA allele frequencies
Shared fraction to European American (EA) allele frequencies
