# Supplementary material for: Affected Kindred Analysis of Human X Chromosome Exomes to Identify Novel X-Linked Intellectual Disability Genes
Source: PLoS One. 2015 Feb 13;10(2):e0116454. doi: 10.1371/journal.pone.0116454 (PMC4332666; doi:10.1371/journal.pone.0116454)
Supplement: S1 Table — describes the probability of observing n number of mutant alleles in the male-only sub-population of the 1000 Genomes project without observation of the mutant allele at all in the female sub-population. S1 Table shows that the probability of observing 12 or more copies of the mutant allele in males only is less than 1%, which commends this allele count as an appropriate cutoff for the minor allele frequency. (PDF) [file pone.0116454.s004.pdf]

Table S1. Calculation of Rare Variant Minor Allele Frequency (MAF) Cutoff

# of ♂ X Chrs (***m***)	# of ♀ X Chrs (***f***)	# of X Chrs Total
525	2 x 567	1659

# of mut. alleles in 1000 Genomes (***n***)	Prob. alleles only in ♀ carriers	Prob. (eq) alleles only in ♀ carriers
1	68.35%	$\prod_{i=(f-n+1)}^{f} \frac{2i}{2i+m}$
2	46.70%	
3	31.88%	
4	21.76%	
5	14.84%	
6	10.11%	
7	6.89%	
8	4.69%	
9	3.19%	
10	2.17%	
11	1.48%	
**12***	**1.00%**	**12 / 1659 = 0.73%**

* At 12 copies of a mutant allele in the 1000 Genomes dataset, the probability of seeing all 12 alleles in only female carriers is only 1%. At >12 copies of a mutant allele, the probability is less than 1%. 12 mutant allele copies is ~ 0.73% minor allele frequency. We can safely assume that potential pathological variants with a MAF < 0.73% could exist purely in a female carrier state. Therefore, such variants should be removed from dbSNP before implemenation of a dbSNP-based filter.
